# RUNX1 expression dynamics in plasma cell differentiation and pathogenesis of multiple myeloma

**DOI:** 10.3389/fimmu.2025.1643615

**Published:** 2025-09-08

**Authors:** Ting Fang Tang, Yee Teng Chan, Hui Jing Lim, Yi Ying Cheok, Nur Adila Anuar, Chin Sum Cheong, Chung Yeng Looi, Sen Mui Tan, Won Fen Wong, Gin Gin Gan

**Affiliations:** ^1^ Department of Medical Microbiology, Faculty of Medicine, Universiti Malaya, Kuala Lumpur, Malaysia; ^2^ Department of Medicine, Faculty of Medicine, Universiti Malaya, Kuala Lumpur, Malaysia; ^3^ School of Biosciences, Faculty of Health & Medical Sciences, Taylor’s University, Subang Jaya, Selangor, Malaysia; ^4^ Digital Health and Medical Advancement Impact Lab, Taylor’s University, Subang Jaya, Selangor, Malaysia; ^5^ Department of Haematology, Hospital Ampang, Jalan Mewah Utara, Pandan Mewah, Ampang, Selangor, Malaysia

**Keywords:** multiple myeloma, plasma cells, RUNX1, B cell differentiation, plasmablast

## Abstract

Multiple myeloma (MM) is characterized by the malignant proliferation of plasma cells within the bone marrow. The transcriptional mechanisms governing plasma cell differentiation and MM pathogenesis are regulated by an intricate network of transcription factors, the role of RUNX1 in this process remains poorly defined. This study aimed to characterize plasma cell subsets in MM and evaluate the expression and functional role of RUNX1 during B cell differentiation. Bone marrow and peripheral blood samples were collected from 61 MM patients and 18 healthy donors. Flow cytometry was used to identify B cell and plasma cell subsets and measure RUNX1 expression across B cell maturation stages. Functional validation was conducted via siRNA-mediated RUNX1 knockdown in CD19^+^ B cells followed by *in vitro* plasma cell differentiation assays. Our data showed that MM patients exhibited significantly increased proportions of plasma cells in bone marrow compared to healthy controls. Intriguingly, RUNX1 expression was low in naive B cell subsets but increased progressively through plasmablast, pre-plasma, and plasma stages. Although RUNX1 expression did not significantly differ across MM stages or between newly diagnosed and relapsed/refractory cases, plasmablasts from MM patients showed higher RUNX1 levels than those from controls. Knockdown of RUNX1 *in vitro* using siRNA delayed B cell differentiation transiently. In summary, RUNX1 expression is dynamically upregulated during terminal B cell differentiation and is elevated in MM-derived plasmablasts. These findings provide new insights into a potential role for RUNX1 in the B cell differentiation axis and MM disease progression.

## Introduction

Multiple myeloma (MM) is one of the most common hematologic malignancies, accounting for approximately 10% of hematopoietic system tumors. It is characterized by the clonal expansion of malignant plasma cells within the bone marrow, resulting in overproduction of monoclonal immunoglobulins, and may present with complications such as renal failure and lytic bone lesions (1). MM progresses through several stages, starting from a premalignant condition known as monoclonal gammopathy of undetermined significance, followed by smoldering MM, active MM, and ultimately but rarely advancing to plasma cell leukemia, the most aggressive and terminal stage of the disease. MM remains an incurable malignancy of plasma cells despite significant advances in diagnosis and treatment over the past two decades. The introduction of proteasome inhibitors, immunomodulatory drugs, monoclonal antibodies, and autologous stem cell transplantation has substantially improved patient outcomes and extended survival (2). However, MM is characterized by inevitable relapse due to the emergence of drug-resistant clones, profound genetic heterogeneity, and disease evolution over time (3). Clonal evolution under therapeutic pressure contributes significantly to treatment failure, particularly in relapsed and refractory MM (RRMM); moreover, the persistence of minimal residual disease (MRD), even following complete remission, presents a critical challenge for achieving a definitive cure (4). The tumor-supportive bone marrow microenvironment, which promotes immune evasion and plasma cell survival, further complicates therapeutic efforts (5, 6). High-risk cytogenetic features such as del (17p), t (4, 14), and 1q gains are associated with poor prognosis and resistance to standard therapies, underscoring the urgent need for personalized treatment strategies and novel therapeutic targets in MM management (7, 8).

Plasma cells arise from B cells that undergo a highly orchestrated differentiation process involving activation in germinal centers, followed by the generation of plasmablasts and their subsequent maturation into antibody-secreting plasma cells (9). Pre-plasmablasts represent an early, transient state that arises shortly after B cell activation. These cells initiate low-level expression of key transcription factors such as IRF4 and BLIMP1 but lack robust immunoglobulin secretion and the complete phenotypic attributes of plasmablasts. They exhibit intermediate expression of activation markers such as CD38 and CD138 and have not yet acquired full effector function (10). Plasmablasts are proliferative, short-lived antibody-secreting cells that represent a critical transitional stage before advancing into terminally differentiated plasma cells. They are typically characterized by high expression of CD38, variable expression of CD138, and reduced expression of B cell markers such as CD20. Functionally, plasmablasts secrete immunoglobulin and retain migratory potential, enabling their movement from secondary lymphoid organs to bone marrow niches for further maturation (11). Further along the pathway, pre-plasma cells represent a more advanced differentiation state that bridges plasmablasts and plasma cells. These cells begin to acquire hallmark plasma cell features—including increased immunoglobulin production and elevated expression of BLIMP1 and XBP1—while still maintaining limited proliferative capacity and some residual expression of early B cell surface markers. Pre-plasma cells are commonly observed in secondary lymphoid tissues and bone marrow during active immune responses, where they likely serve as an intermediate reservoir for plasma cell development (9, 12).

The process of plasma cell differentiation is regulated by a tightly controlled transcriptional network involving key factors. Interferon regulatory factor 4 (IRF4) acts as a central node, dynamically controlling the switch between germinal center B cells and plasma cells, depending on its expression levels (13, 14). B lymphocyte-induced maturation protein 1 (BLIMP1, encoded by PRDM1) serves as the master regulator of plasma cell fate by repressing genes involved in the B cell program, including paired box gene 5 (PAX5) and B-cell lymphoma 6 protein (BCL6), and upregulating those essential for plasma cell function, such as X-box binding protein 1 (XBP1), which drives immunoglobulin production and the unfolded protein response (15, 16). In contrast, B-cell lymphoma 6 protein (BCL6) and basic leucine zipper transcription factor 2 (BACH2) maintain B cell identity and germinal center function by repressing BLIMP1 expression and must be downregulated for plasma cell commitment to occur (17).

The Runt-related transcription factor (RUNX) family comprises three core members, namely RUNX1, RUNX2, and RUNX3, each playing essential and distinct roles in lineage-specific development and cellular differentiation. These transcription factors share a conserved Runt homology domain, which mediates DNA binding and heterodimerization with the cofactor CBFβ (core-binding factor beta), enhancing their transcriptional stability and function (18). RUNX1, also known as AML1, is best known for its critical function in definitive hematopoiesis (19, 20) and lymphocyte development (21), and its frequent mutations are strongly implicated in leukemogenesis, particularly in acute myeloid leukemia (AML) (18, 22). RUNX2 is the master regulator of osteoblast differentiation and is essential for skeletal development; mutations in RUNX2 are associated with cleidocranial dysplasia (23). RUNX3, on the other hand, is involved in the development of dorsal root ganglia, gastric epithelium, and T cell function (24–26). It has been identified as a tumor suppressor in various epithelial cancers, including gastric and lung cancer, where its inactivation leads to aberrant cell proliferation and impaired apoptosis.

RUNX1 has been implicated in the early development of both T and B cells (27–29). In naive T cells, RUNX1 plays a crucial role in maintaining cell quiescence as T cell specific deletion of RUNX1 leads to T cell hyperactivation and autoimmunity in mouse model (30, 31). Despite its involvement in early B cell development, the role of RUNX1 in B cell terminal differentiation and plasma cell identity remains incompletely defined. RUNX1 is expressed in multiple isoforms including RUNX1a, RUNX1b, and RUNX1c, that are generated by alternative promoter usage and splicing (32). RUNX1b is the dominant functional isoform involved in lymphocyte development and gene regulation. RUNX1c is mainly expressed during definitive hematopoietic stem cells development, while RUNX1a may act as a dominant-negative regulator by competing for DNA binding without initiating transcription.

Given that MM arises from long-lived plasma cells, we hypothesize that RUNX1 may influence key transitional states during B cell differentiation and plasma cell commitment. In this study, we investigated the expression profile of RUNX1 across various B cell subsets and plasma cell stages in MM patients and healthy donors.

## Methods

### Study participants

A total of 61 individuals who were diagnosed with MM were recruited to this study from the Universiti Malaya Medical Centre (UMMC) and Hospital Ampang, Kuala Lumpur from 2018 to 2022. This study has obtained ethical approval from Universiti Malaya Research Ethics Committee (MREC ID No: 2018116-5966) and National Medical Research Register (NMRR-18-1439-41302). Written informed consent was obtained from each volunteer and patients prior to sample collection. Healthy individuals are bone marrow stem cell donors, who consented to provide additional samples during marrow collection for this study.

The criteria for the diagnosis, staging and risk status of MM were in accordance with the ISS by the International Myeloma Working Group (IMWG) (33). ISS stage I refers to those with beta-2 microglobulin (B2M) level <3.5 mg/L and serum albumin ≥3.5 g/dL; ISS stage II refers to the patients with B2M <3.5 mg/L and serum albumin <3.5 g/dL, or B2M levels of 3.5 to 5.5 mg/L irrespective of serum albumin level; and ISS stage III refers to those with B2M >5.5 mg/L. Stage 0 refers to those with newly diagnosed solitary plasmacytoma.

### Sample processing

A total of 10 mL of peripheral blood samples and 10 mL of bone marrow aspirates were collected from MM patients and healthy donors. Peripheral blood samples were diluted at 1:2 while bone marrow aspirates were diluted at 1:7 ratio with phosphate buffer saline (PBS), before layered over 10 mL lymphocyte separation medium (Corning^®^, Corning, New York, USA) and centrifuged at 400× g for 30 minutes at room temperature. The buffy coat layers containing peripheral blood mononuclear cells (PBMCs) or bone marrow mononuclear cells (BMMCs) were collected in a 10 mL tube and washed twice with PBS solution. Cells were resuspended in human serum containing 5% dimethyl sulfoxide (DMSO) and stored in liquid nitrogen tank until further analysis.

### Immunophenotyping and intracellular staining

The cryopreserved PBMCs and BMMCs were retrieved from liquid nitrogen tank and thawed in water bath at 37°C. The cells were pelleted at 300 ×g for 5 minutes at 4°C and washed using PBS/3%FBS. The cells were incubated with staining for extracellular markers for 30 minutes at 4°C using the following fluorochromes: APC anti-human CD19, Alexa Fluor 488 anti-human CD20, PerCP-Cyanine 5.5 CD27, APC-Cyanine 7 CD38 and PE-Cyanine 7 CD138 (BioLegend, San Diego, USA), with dilution factor of 1:200. After 30 minutes of incubation, cells will be washed and resuspended with 1 mL of PBS/3%FBS.

Following extracellular surface staining, cells were fixed and permeabilized using the Foxp3/Transcription Factor Staining Buffer Kit (Invitrogen, Stockholm, Sweden). After washing, the cell pellet was resuspended in 1 mL of fixation/permeabilization buffer and centrifuged at 300 ×g for 5 minutes at 4°C. The supernatant was discarded, and the cells were then stained intracellularly with PE-conjugated anti-RUNX1 (eBiosciences, Thermo Fisher Scientific, Waltham, Massachusetts, USA) for 30 minutes in dark. PE-anti-RUNX1 is a monoclonal antibody (clone RXDMC) specific for RUNX1 protein. Cells were washed and resuspended in 200 µL of PBS/3%FBS. Cells were examined in a BD FACSCanto™ II flow cytometer, and analyzed using BD FACSDiva™ and FlowJo softwares (BD Biosciences).

### RNA isolation and cDNA synthesis

Cells were resuspended in 1 mL of TRIzol™ reagent (Invitrogen, Carlsbad, California, USA) and incubated at room temperature for 5 minutes. Subsequently, 200 µL of chloroform were added, vortexed briefly, and incubated for 2 to 3 minutes at room temperature. Samples were centrifuged at maximum speed at 4 °C for 15 minutes, and the upper aqueous phase was carefully transferred to a new tube. Next, 500 µL of 100% isopropanol were added, mixed gently, incubated at room temperature for 10 minutes, and centrifuged at 4 °C for 10 minutes. The RNA pellet was washed with 1 mL of 75% ethanol, air-dried at room temperature for 1 minute, and dissolved in 12 µL of RNase-Free Water. The dry RNA pellet was dissolved and measured using NanoDrop™ 2000 spectrophotometers (Thermo Scientific, Waltham, Massachusetts, USA) at 260nm and 280nm to determine sample concentration and purity. A total 1μg of RNA was used for cDNA synthesis using the SuperScript™ IV First-Strand Synthesis System (Invitrogen). The reaction mixture contained random hexamers, dNTPs, RNaseOUT™ inhibitor, and SuperScript™ IV reverse transcriptase was performed under the following thermal cycling conditions: primer annealing at 65°C for 5 min, extension at 50°C for 10 min, and enzyme inactivation at 80°C for 10 min. The resulting cDNA was stored at −20°C. All the primers used were designed using Primer-BLAST.

### qRT-PCR

Quantitative real time polymerase chain reaction (qRT-PCR) analysis was carried out using SsoAdvanced™ Universal SYBR Green Supermix (Bio-Rad, Hercules, California, USA). Primers used were as follow: *Runx1* (5’-AACCTCGAAGACATCGGCAG-3’ and 5’-AAGGCAGTGGAGTGGTTCAG-3’) detecting all RUNX1 isoforms, *RUNX1a* (5’-ATGCGTATCCCCGTAGATGC-3’ and 5’-CATGGCTGCGGTAGCATTTC-3’), *RUNX1b* (5’-CGACTCTCAACGGCACCCGA-3’ and 5’-ATGGCCGACATGCCGATGCC-3’), *RUNX1c* (5’-GGCCTCATAAACAACCACAGA-3’ and 5’-CTGTGGGTACGAAGGAAATGA-3’), *BCL6* (5’-GGACTCCACCATCCCACAAG-3’ and 5’-AGTGTGGGTTTTCAGGTTGGCT-3’), *BACH2* (5’-AGCAGAGAAAACATCCGCGAG-3’ and 5’-GGCTTGAGGCTGTTGCTAGA-3’), *PAX5* (5’-GTCCATTCCATCAAGTCCTG-3’ and 5’-TTGCTGACACAACCATGGCT-3’), *IRF4* (5’-TTCCGAGAAGGCATCGACAAG-3’ and 5’-GCAGACCTTATGCTTGGCTC-3’), *BLIMP1* (5’-ACCCAGTTTGTGCACCTGAAAC-3’ and 5’-CAGAGGTAGTGGGTTGCTGG -3’), *XBP1* (5’- TAAGACAGCGCTTGGGGATGG -3’ and 5’-GGCTGGTAAGGAACTGGGTC-3’), and *GAPDH* (5’- CATGACCACAGTCCATGCCATCACT-3’ and 5’-TGAGGTCCACCACCCTGTTGCTGTA-3’). Housekeeping gene *GAPDH* was used for normalization to obtain relative fold changes based on comparative threshold cycle (ΔΔCt) method. All reactions were run in duplicate in the Agilent Mx3000P™ qPCR system (Agilent Technologies, Santa Clara, California, USA). Condition is as follow: 95˚C for 2 minutes, 40 cycles of 95˚C denaturation for 15 seconds, 60˚C annealing for 1 minute and 60˚C extension for 1 minute, following by dissociation.

### B cell isolation

PBMCs were isolated from buffy coats of healthy donors’ peripheral blood samples by density gradient centrifugation using LSM and washed twice in PBS. Cell suspensions were resuspended in complete RPMI medium supplemented with 10% FBS. Human CD19^+^ B cells were isolated using the BD IMag™ anti-human CD19 Particles – DM. A total of 50 µl of the particles was added per 1 × 10^7^ cells, incubated for 10 minutes and washed. After two rounds of isolation, cell pellet was resuspended in complete RPMI media with 10% FBS. Purity of the post-sorted CD19^+^ B cell was >97%.

### siRNA knockdown and *in vitro* plasma cell induction

Control-siRNA-A (sc37007) and specific RUNX1-siRNA (sc-37677) (Santa Cruz Biotechnology, Dallas, Texas, USA) were commercially acquired. and A total of 8 µL of siRNA (140 ng/µL) and 12 µL of 6X Lipofectamine 3000 were diluted in 5.6 mL of RPMI and incubated at room temperature for 15 minutes. The transfection solution was prepared by adding 200 µL of the reagent mixture into 0.8 mL of blank RPMI. Cells were resuspended in the transfection solution and seeded into a 6-well plate at a final concentration of 2.5 ×10^5^ cells per mL. The cells were incubated for 5 hours, followed by replacement with RPMI supplemented with 10% FBS, and further incubated for 18 hours in a CO_2_ incubator. The transfection process was repeated twice. Subsequently, cells were cultured in RPMI medium containing 500 ng/mL sCD40L and 20 ng/mL IL-4. On day 3, the cells were transferred to RPMI medium containing 500 ng/mL sCD40L and 10 ng/mL IL-21. The medium was replenished with fresh medium every 2 days, and cells were harvested for analysis on day 11.

### Statistics

Differences between groups were analyzed by Student’s *t*-test or Mann-Whitney U-test in GraphPad Prism v10. Statistical significance is indicated with *P < 0.05, **P < 0.01, ***P < 0.001, and ****P < 0.0001.

## Results

### Patient demography

A total of 61 MM patients and 18 healthy donors were recruited in this study, as summarized in **Table 1**. Among MM cohort, 70% (43/61) were newly diagnosed multiple myeloma (NDMM) while the remaining 23% (14/61) were relapsed and refractory multiple myeloma (RRMM). The patient cohort was further categorized according to ISS into different stages: 16% (10/61) stage I, 10% (6/61) stage II, and 62% (38/61) stage III, whereas no staging were reported for 4 patients due to incomplete information. The age for the MM patients and healthy individuals ranged between 34 to 80 years old and 21 to 69 years old, respectively. Among the MM patients, 59% (36/61) were male and 41% (25/61) were female while the cohort comprised 54.1% (33/61) Malay, 32.8% (20/61) Chinese and 13.1% (8/61) Indian ethnicity.

**Table 1:** Patient demography.

	MM n=61	Controls n=18	P value	OR (95% CI)
Gender				
Male	59% (36/61)	39% (7/18)	0.1796	2.263 (0.8148-6.117)
Female	41% (25/61)	61% (11/18)		
Age				
≤ 55 y/o	16	16	<0.0001	0.0444 (0.0097-0.2083)
> 55 y/o	45	2		
Range	34 - 80	21 - 69		
Ethics				
Malay	54.1% (33/61)	16.7% (3/18)	0.0066	5.893 (1.655-20.32)
Chinese	32.8% (20/61)	77.8% (14/18)	0.0010	0.1394 (0.0465-0.4510)
Indian	13.1% (8/61)	5.5% (1/18)	0.6757	2.566 (0.4170-30.00)
Serum albumin level, g/L				
Low	28			
Normal	30			
High	3			
β2-microglobulin, mg/L				
Normal	10			
High	37			
No information	14			
Disease type				
Newly diagnosed MM	43			
Relapsed/refractory MM	14			
Non-secretory MM	1			
Plasmacytoma	3			
Heavy chain isotype				
IgG	33			
IgA	8			
IgM	1			
Light chain				
Light chain isotype	3			
Kappa	7			
Lambda	4			
International staging (ISS)				
Stage I	10			
Stage II	6			
Stage III	38			
No staging	4			

Characteristics of 61 Multiple myeloma patients and 18 healthy donors recruited. Statistical analysis was performed using Fisher’s exact test. MM: multiple myeloma, OR: odd-ratio, CI: confidence interval, y/o: years old.

Given the age difference between the control and MM cohorts, additional analyses were performed to assess whether age influences B cell subset distribution among MM patients. Scatter plots examining the correlation between age and the frequency of individual plasma cell subsets, including early, intermediate, and mature populations, revealed no significant associations (**Supplementary Figures S1** and **S2**). These findings suggest that age does not substantially impact the proportional distribution of plasma cell subsets within the MM cohort.

### High percentages of plasma cells in MM bone marrow

**Figure 1:**
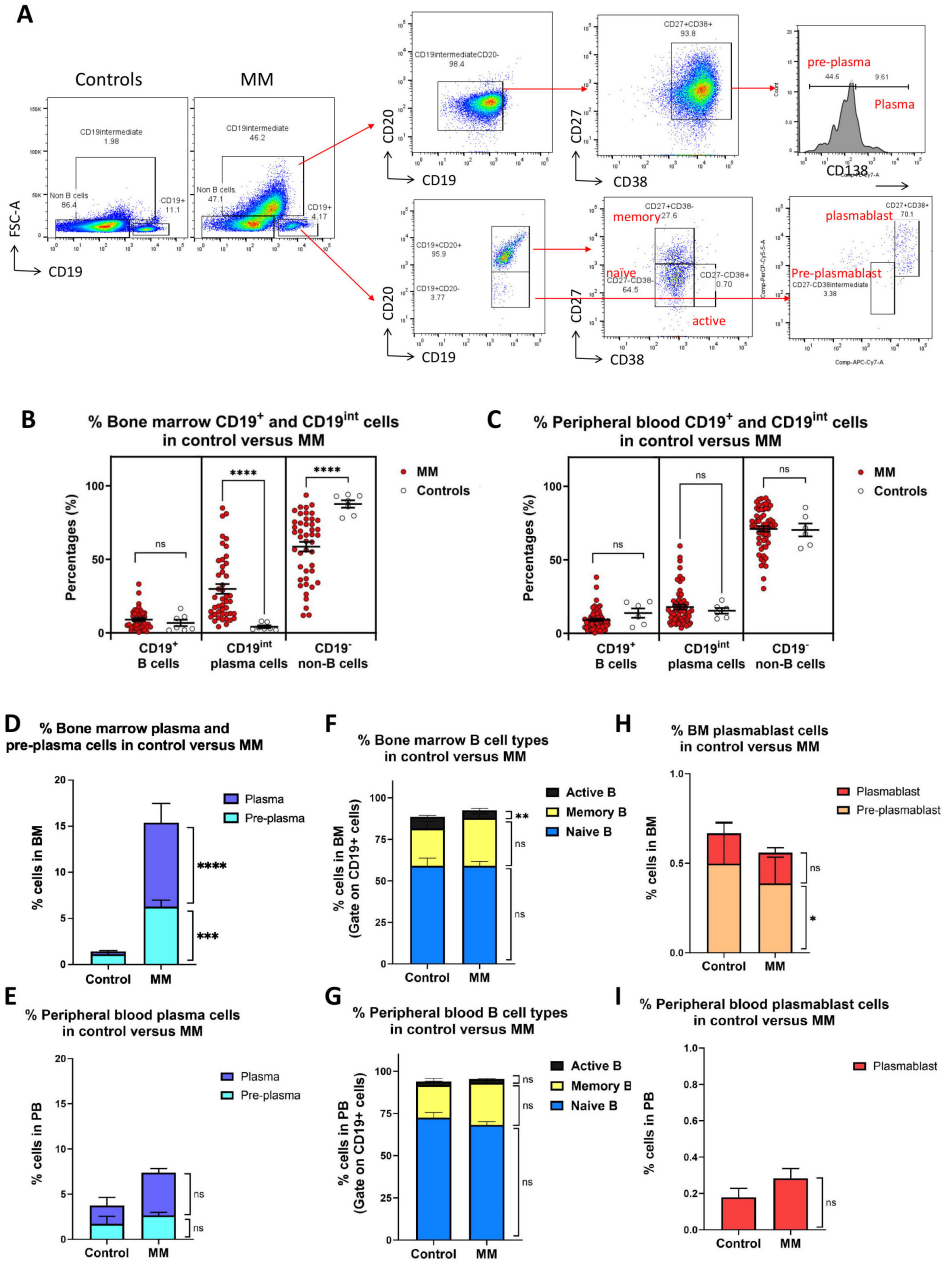
Immunophenotyping of bone marrow and peripheral blood cells mononuclear cells in MM versus healthy controls. **(A)** Gating strategies for different B cell populations in MM bone marrow and peripheral blood. Three distinct populations were first gated in control and MM including B (CD19^+^FCS^low^), differentiated cell (CD19^int^FCS^high^) and non-B cell (CD19^-^FCS^low^). CD19^int^FCS^high^ population was further divided into pre-plasma (CD19^int^CD20^-^CD27^+^CD38^+^CD138^low^) and plasma cell (CD19^int^CD20^-^CD27^+^CD38^+^CD138^high^). CD19^+^ B cells were further subdivided into CD20^+^ and CD20^-^ cells. CD20^+^ population include naïve (CD19^+^ CD20^+^ CD27^-^ CD38^-^), active (CD19^+^ CD20^+^ CD27^-^ CD38^+^), and memory (CD19^+^ CD20^+^ CD27^+^ CD38^-^) B cells, whereas CD20^-^ population include pre-plasmablasts (CD19^+^CD20^-^CD27^-^CD38^int^), plasmablasts (CD19^+^CD20^-^CD27^+^CD38^+^). **(B, C)** Dot plot shows the percentages of B (CD19^+^FCS^low^), plasma cell (CD19^int^FCS^high^) and non-B cell (CD19^-^FCS^low^) in the bone marrow and peripheral blood derived from MM (n=45) versus controls (n=7). Each dot represents one individual. **(D, E)** Bar charts show the percentages of plasma and pre-plasma cell in bone marrow or peripheral blood derived from MM versus controls. **(F, G)** Bar charts show the percentages of naive, memory and active B cells cell in bone marrow or peripheral blood derived from MM versus controls. **(H, I)** Bar charts show the percentages of pre-plasmablast and plasmablast cells in the bone marrow or peripheral blood derived from MM versus controls. Data are shown as mean ± SEM. Statistical analysis by non-parametric Mann-Whitney U test. ****P < 0.0001; ns, not significant.

Bone marrow and peripheral blood samples from MM patients and healthy controls were first immunophenotyped using flow cytometry (**Figure 1A**; left panel). In bone marrow derived from healthy controls, two major cell populations were detected using FSC and CD19 marker, i.e. CD19^+^ B cells and CD19^-^ non-B cells. Whereas in MM bone marrow, one extra distinct cell population was identified, i.e. CD19^int^ FCS^high^ cells, in addition to B and non-B populations. In bone marrow, the percentages of CD19^int^ FCS^high^ cells were significantly elevated in MM patients (29.9 ± 3.3%) compared to controls (4.2 ± 0.9%; P < 0.0001) (**Figure 1B**). The increase in CD19^int^ FCS^high^ cells was accompanied by a marked decrease in CD19^-^ non-B cells in MM patients (58.6 ± 3.3%) compared to controls (87.7 ± 2.5%; P < 0.0001), whereas the percentages of CD19^+^ B cells did not differ significantly between MM patients (9.0 ± 1.0%) and controls (6.8 ± 2.2%; P = 0.3405). In peripheral blood, the average percentages of CD19^+^ B cells were similar between MM patients (9.1 ± 0.9%) versus controls (13.8 ± 3.2%; P = 0.1340) (**Figure 1C**). The mean percentage of CD19^int^ FCS^high^ plasma cells was comparable in MM patients (17.9 ± 1.6%) compared to controls (15.4 ± 2.0%; P=0.9017). Similarly, no significant difference was observed in the percentage of CD19^-^ non-B cells between MM patients (71.1 ± 1.9%) and controls (70.4 ± 4.4%; P = 0.7276). These results underscore a notable expansion of CD19^int^ FCS^high^ population in the bone marrow of MM patients.

Using surface identity markers, CD19^int^ FCS^high^ cells were further subcategorized into pre-plasma cells (CD19^int^ FCS^high^ CD20^-^ CD27^+^ CD38^+^ CD138^low^) and plasma cells (CD19^int^ FCS^high^ CD20^-^ CD27^+^ CD38^+^ CD138^high^) (**Figure 1A**; upper right panel). The percentages of both bone marrow pre-plasma and plasma cells were significantly elevated in MM cohort compared to controls (**Figure 1D**). The average percentage of pre-plasma cells was approximately 6 folds higher in MM (6.3 ± 0.7%) than in controls (1.1 ± 0.2%; P = 0.0001). Similarly, the percentage of plasma cells was significantly increased by 3 folds in MM patients (9.1 ± 2.1%) compared to controls (0.3 ± 0.1%; P < 0.0001). In peripheral blood, no statistically significant differences were observed for pre-plasma and plasma cells. Percentages of plasma cells showed an approximately 2.5-fold increase in MM (4.7 ± 0.5%) compared to controls (2.0 ± 0.9%; P=0.0526), whereas percentages of pre-plasma cells remain unchanged between MM patients (2.7 ± 0.3%) and controls (1.7 ± 0.8%; P = 0.2633) (**Figure 1E**).

CD19^+^ B cells were further subdivided into different subsets: naïve B cells (CD19^+^ CD20^+^ CD27^-^ CD38^-^), active B cells (CD19^+^ CD20^+^ CD27^-^ CD38^+^), and memory B cells (CD19^+^ CD20^+^ CD27^+^ CD38^-^) (**Figure 1A**; lower right panel). In the CD19^+^–gated population of bone marrow samples, no apparent differences were observed in the composition of naïve or memory B cell subsets between MM patients and controls. The average percentage of naïve B cells was comparable in MM (59.0 ± 2.7%) compared to controls (59.1 ± 4.6%; P = 0.8359) (**Figure 1F**). Likewise, the percentages of memory B cells were comparable between MM patients (28.8 ± 2.5%) and controls (22.5 ± 4.1%; P = 0.3745). However, a significant reduction in the percentages of active B cells was observed in MM patients (4.7 ± 1.2%) compared to controls (7.0 ± 0.8%; P = 0.0063). In peripheral blood, no significant differences were noted across all three CD19^+^ B cell subsets (**Figure 1G**). The percentages of naives B cells were comparable between MM patients (68.2 ± 1.9%) and controls (72.5 ± 3.2%; P = 0.5697). Similarly, the percentages of memory B cells were not significantly different between MM patients (25.0 ± 2.0%) and controls (19.3 ± 3.9%; P = 0.4474). The average percentages of active B cells was also dissimilar in MM patients (2.1 ± 0.4%) compared to that of controls (2.1 ± 0.3%; P = 0.0995). The percentages of both pre-plasmablast and plasmablast cells were generally reduced in the MM cohort compared to controls. In bone marrow, the average percentage of pre-plasmablast cells was slightly lower in MM patients (0.4 ± 0.1%) compared to controls (0.5 ± 0.2; P = 0.0499) (**Figure 1H**). Similarly, the proportion of plasmablast cells was comparable between MM patients (0.2 ± 0.0%) and controls (0.2 ± 0.1%; P = 0.7931). In peripheral blood, no pre-plasmablast cell population was detected in either group. There were no significant differences in plasmablast cell percentages between MM patients (0.3 ± 0.1%) and controls (0.2 ± 0.0; P = 0.6415) (**Figure 1I**).

### Elevated RUNX1 expression as B cells advance into plasma cells

**Figure 2:**
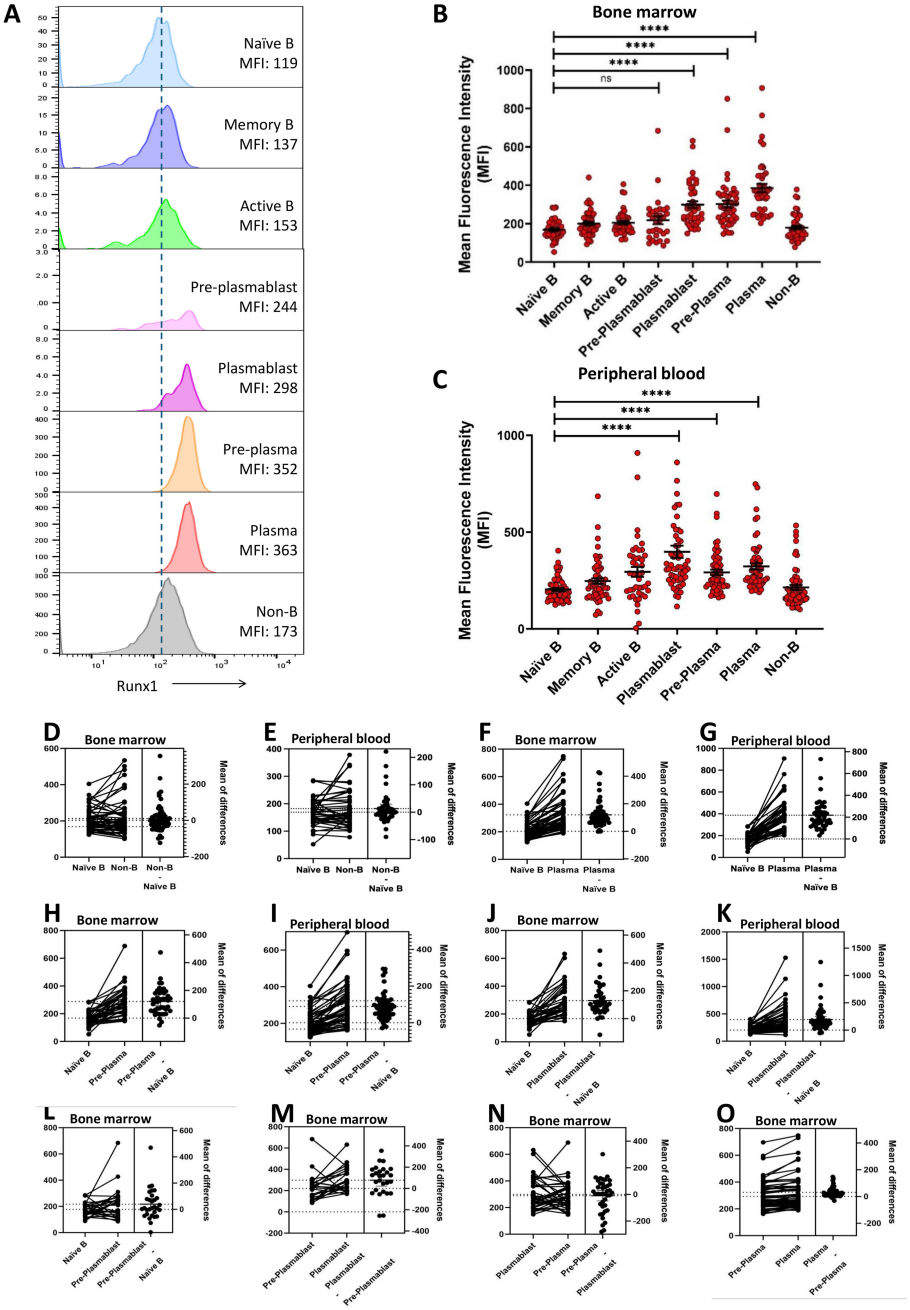
Elevated RUNX1 expression in the terminally differentiated plasmablast and plasma cells. **(A)** Representative histograms of RUNX1 expression across various cell types in bone marrow of an MM patient (M59M). A gradual increase in mean fluorescent intensity (MFI) of RUNX1 was observed as naïve B cells differentiate into plasma cells during the plasma cell. **(B, C)** Dot plot shows the mean fluorescent intensity (MFI) of RUNX1 in different B cell differentiation stages in the bone marrow and peripheral blood derived from MM patients. Elevated expression of RUNX1 can be observed from plasmablast stage which was peaked at plasma cell stage. Each dot represents one individual, and data is shown as mean SEM. Statistical analysis by non-parametric Mann-Whitney U test. ****P < 0.0001; ns, not significant. **(D-K)** Estimation plots of comparison for RUNX1 MFI between different B cell subsets in bone marrow or peripheral blood from the MM patients. **(L-O)** Estimation plots of stepwise comparisons of each transitional stage when MM bone marrow B cells progressed from pre-plasmablast to plasmablast, pre-plasma to plasma cell.

As B cells differentiate into plasma cells, various molecules are involved in the differentiation process. We subsequently analyzed the expression profile of RUNX1 across different B cell subsets and observed a notable increase in RUNX1 expression during the transition from B cells to plasma cells (**Figure 2A**). RUNX1 expression was relatively low in non-B cells, naïve B cells, memory B cells, and activated B cells. However, its expression markedly increased in pre-plasmablasts and continued to rise in plasmablasts. The highest levels of RUNX1 were observed in pre-plasma and plasma cells.

In bone marrow cells derived from MM patients, naïve B cells (169 ± 7), memory B cells (200 ± 10), and activated B cells (206 ± 10) exhibited low expression or mean fluorescent intensity (MFI) of RUNX1, comparable to that of non-B cells (214 ± 13) (**Figure 2B**). RUNX1 expression remained low at the pre-plasmablast stage (219 ± 21), but increased notably in plasmablasts (299 ± 18). A further significant upregulation was observed in pre-plasma (292 ± 14) and plasma cells (323 ± 16), indicating progressive induction of RUNX1 during terminal B cell differentiation. A similar trend was observed in peripheral blood-derived cells, where RUNX1 expression was low in naïve B cells (169 ± 7) and elevated in pre-plasma (292 ± 14) and plasma cells (323 ± 16) (**Figure 2C**). Notably, the highest RUNX1 expression was detected in plasmablasts (398 ± 31) in MM peripheral blood.

Estimation plot analysis demonstrated no marked differences between naive B and non-B cells in both bone marrow and peripheral blood (**Figures 2D, E**). A marked increase in RUNX1 expression during the transition from naïve B cells to plasma cells, pre-plasma and plasmablast (**Figures 2F–K**). This trend was consistently observed in paired samples from the same individuals, across both peripheral blood and bone marrow. In contrast, comparison between naïve B cells and early pre-plasmablast cells did not reveal any significant change in RUNX1 expression (**Figure 2L**). Other stepwise comparisons of each transitional stage were conducted. As cells progressed from pre-plasmablast to plasmablast, an increasing trend was observed (**Figure 2M**). However, this trend was not evident in the later stages, this trend was not evident in the later stages when pre-plasma cell advanced into plasma cell (**Figures 2N, O**). These findings collectively suggest a potential role for RUNX1 in plasma cell differentiation, particularly during the early transition of pre-plasmablast to plasmablast stage.

### RUNX1 expression in plasma cell differentiation in MM versus controls

**Figure 3:**
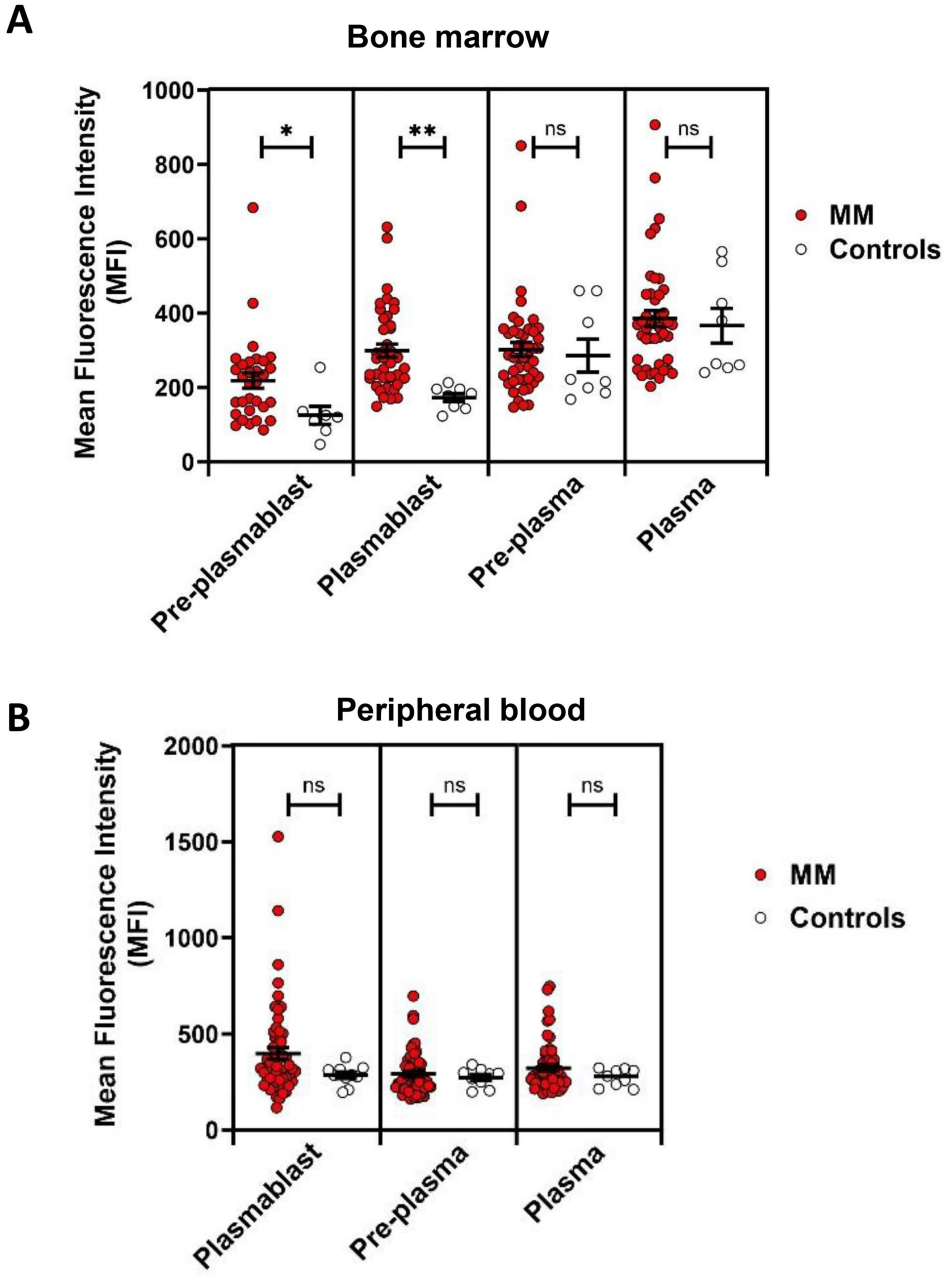
RUNX1 expression in plasmablasts and plasma cells in MM versus healthy controls. **(A)** Dot plots show the MFI of RUNX1 expression in pre-plasmablast, plasmablast, pre-plasma and plasma cells in bone marrow in the MM versus healthy controls. A higher expression of RUNX1 can be seen in the MM preplasmablast and plasmablast cells, compared to the healthy controls. However, the pre-plasma or plasma cells from MM shows comparable level of RUNX1 when compared to the controls. **(B)** No significant differences can be seen in the peripheral blood. Note that pre-plasmablast population does not exist in the peripheral blood. Each dot represents one individual, and data is shown as mean SEM. Statistical analysis by non-parametric Mann-Whitney U test (*P < 0.05; **P < 0.01; ns, not significant).

MM is characterized by expansion of malignant plasma cell in bone marrow. We next examined if the RUNX1 expression differs in healthy control and MM patients (**Figure 3A**). However, no significant differences were detected in RUNX1 expression in bone marrow plasma cells derived from MM (386 ± 22) versus controls (366 ± 47; P = 0.9475). Further investigation revealed significant elevation of RUNX1 expression in bone marrow plasmablasts derived from MM (299 ± 18) compared to healthy controls (173 ± 11; P < 0.0001). In peripheral blood, plasmablasts also displayed a higher RUNX1 levels in MM patients (398 ± 31) compared to controls (286 ± 17), though the difference did not reach statistical significance (P = 0.1441) (**Figure 3B**). While no significant differences were detected in peripheral plasma cells derived from MM (323 ± 16) versus controls (273 ± 15; P = 0.4328).

### RUNX1 expression across MM stages and in NDMM versus RRMM

**Figure 4:**
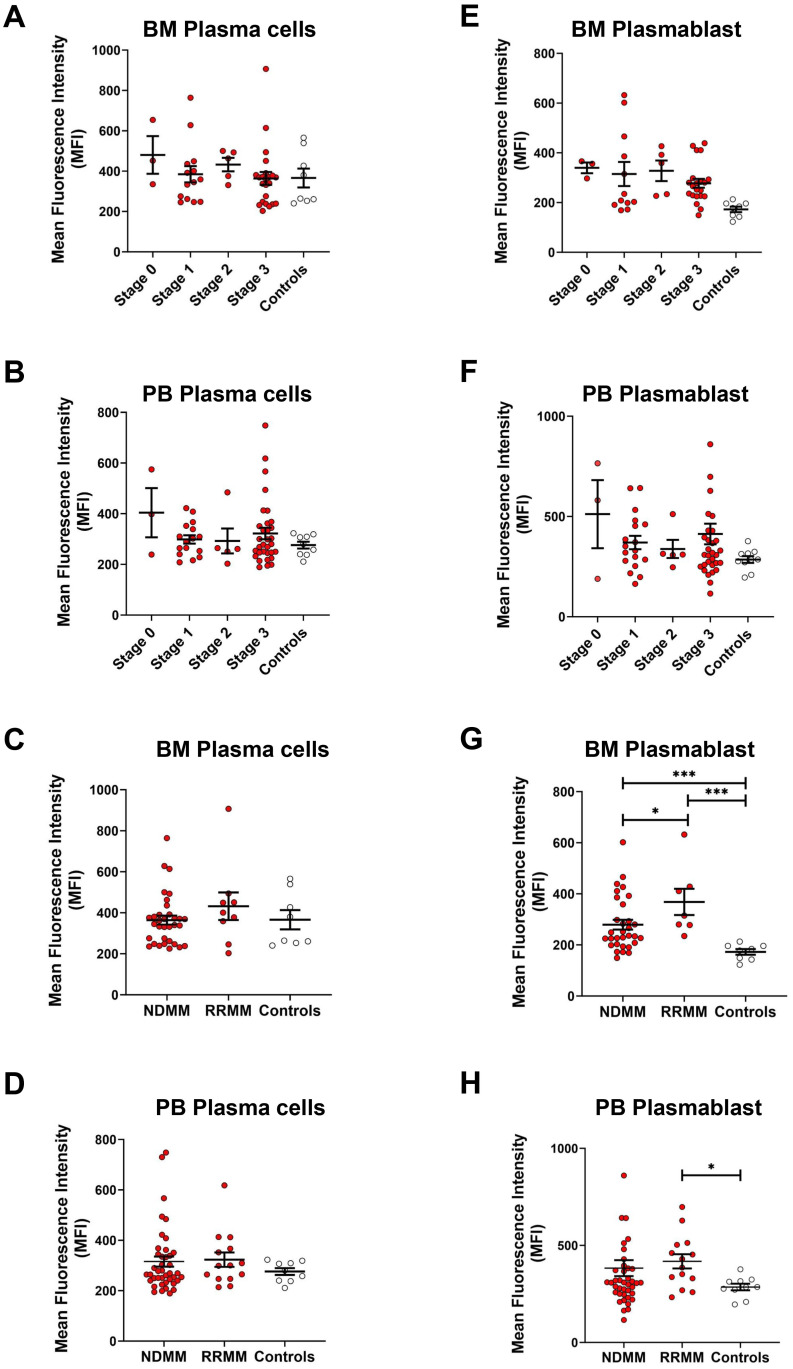
RUNX1 expression in plasma cells in different MM stages and in RRMM versus NDMM. **(A, B)** Dot plots show the MFI of RUNX1 expression in plasma cell populations of different ISS staging in MM bone marrow (BM) and peripheral blood (PB). **(C, D)** Dot plots show the MFI of RUNX1 expression in plasma cells from the NDMM or RRMM, compared to the controls. No significant differences observed in the RUNX1 expression in the plasma cells when comparing different MM stages or disease types. **(E, F)** Dot plots show the MFI of RUNX1 expression in plasmablasts of different ISS staging in MM bone marrow and peripheral blood. **(G, H)** Dot plots show the MFI of RUNX1 expression in plasmablasts from NDMM or RRMM, compared to the controls. Significant differences among these comparisons, as RUNX1 expreesion was high in RRMM. Each dot represents one individual, and data is shown as mean SEM. Statistical analysis by non-parametric Mann-Whitney U test (*P < 0.05; ***P < 0.001; not significant if no asterisks).

RUNX1 expression in plasma cell populations across different MM stages was investigated to determine stage-dependent variations (**Figure 4A**). We noted that RUNX1 expression remained relatively constant across MM stages in bone marrow. The MFI of RUNX1 was recorded as stage 0 (480 ± 93), stage 1 (385 ± 41), stage 2 (433 ± 33) and stage 3 (364 ± 32), compared to controls (366 ± 47). Similarly, no difference was noted when examining RUNX1 expression in peripheral cells across different MM stages (**Figure 4B**). When comparing between NDMM and RRMM patients, there were no significant differences detected either in cells derived from bone marrow (**Figure 4C**) or peripheral blood (**Figure 4D**).

When analyzing the plasmablast population, RUNX1 expression remained relatively similar across different MM stages, with MFI values recorded as follows: stage 0 (340 ± 22), stage 1 (331 ± 49), stage 2 (328 ± 41), and stage 3 (277 ± 18), but consistently elevated when compared to healthy controls (173 ± 11) in bone marrow (**Figure 4E**). Similarly, peripheral blood analysis revealed no marked differences in RUNX1 expression across MM stages (**Figure 4F**), with MFI values of stage 0 (512 ± 170), stage 1 (370 ± 33), stage 2 (338 ± 45), and stage 3 (413 ± 51), but relatively higher versus controls (286 ± 17). When comparing the plasmablast population among NDMM and RRMM, a significant increase in RUNX1 expression was observed in bone marrow-derived plasmablasts (**Figure 4F**). RUNX1 MFI in NDMM patients was 280 ± 19 (P = 0.0004), and in RRMM patients was 368 ± 52 (P = 0.0003), both significantly higher than controls (173 ± 11). A significantly higher RUNX1 was also noted in RRMM compared to NDMM group (P = 0.0431), suggesting a potential association between elevated RUNX1 expression and disease relapse or progression of MM (**Figure 4G**). Peripheral blood samples also showed higher RUNX1 expression in RRMM patients (418 ± 37) compared to NDMM (384 ± 41, P = 0.3891) and controls (286 ± 17, P = 0.0107) (**Figure 4H**).

To further assess whether high RUNX1 expression in plasma cells correlates with donor age, linear regression analysis was performed. The results showed no statistically significant correlation between age and RUNX1 expression across naïve, activated, and differentiated B cells, derived from bone marrow or peripheral blood from healthy donors and MM patients (**Supplementary Figure S3**, **S4**).

#### Transient RUNX1 interference delays *in vitro* plasma cell stimulation

**Figure 5:**
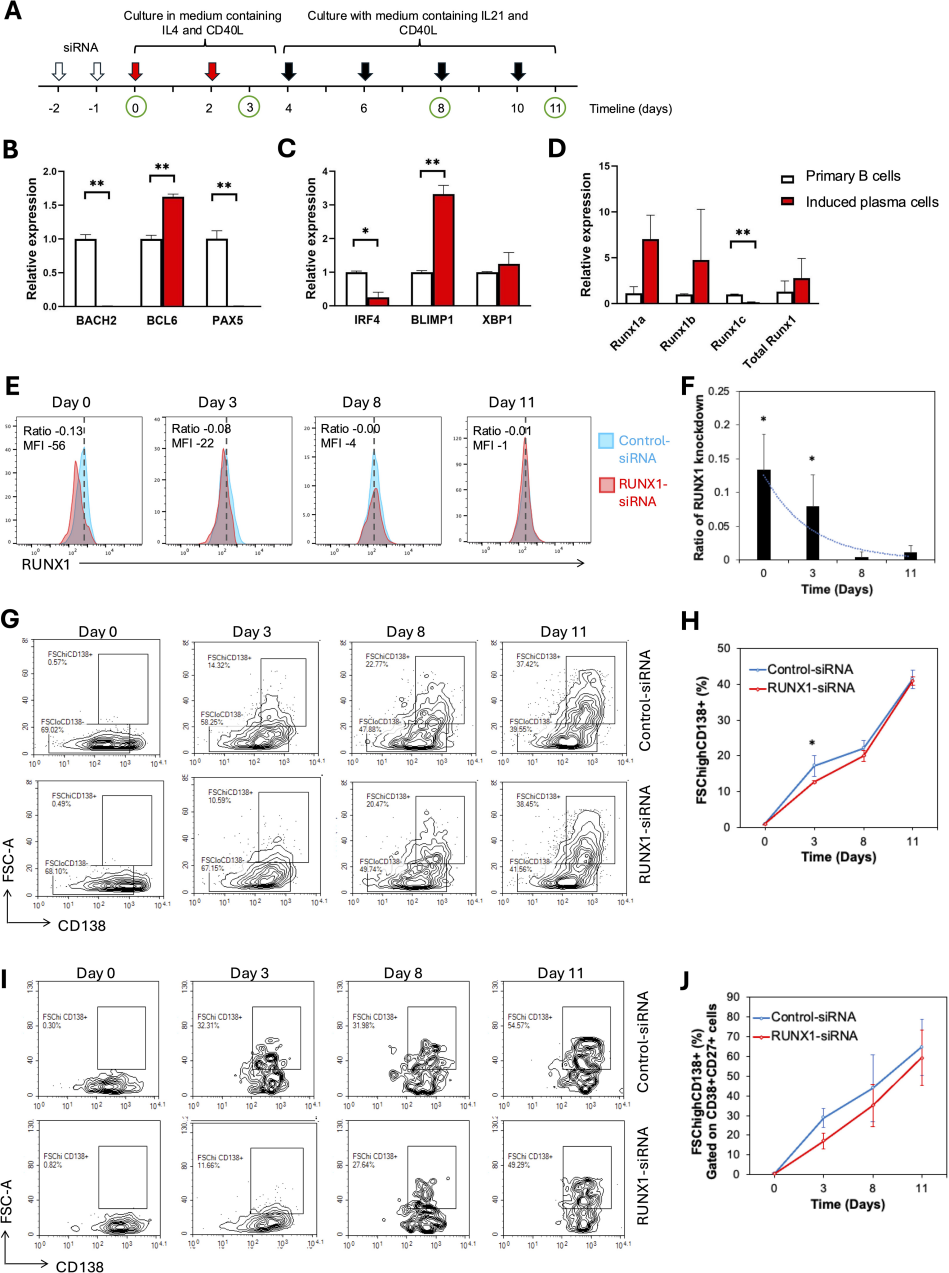
RUNX1 interference in induced plasma cells progression. **(A)** Schematic representation of the experimental workflow for plasma cell induction. Primary CD19^+^ B cells were isolated and cultured in the presence of CD40L, IL-4 and IL-21 for 12 days to induce plasma cell differentiation *in vitro*. From days 0 to 3, cells were stimulated with IL-4 and sCD40L to induce B cell activation and proliferation. From day 4 onward, IL-21 and sCD40L were added to promote terminal differentiation into plasma cells. Fresh cytokines were replenished every two days throughout the culture period. White arrows mark the two rounds of siRNA transfection performed within the first two days. Red arrows indicate replenishments with culture medium containing IL-4 and soluble CD40L, whereas black arrows indicate replenishments with medium containing IL-21 and soluble CD40L during the 11-day culture period. Green circles represent time points for sample collection and analysis. **(A-C)** Bar chart illustrates the relative expression of B cell identity genes in the uninduced primary B (day 0) and differentiating B cells (day 11) in healthy donors. **(B-D)** Real-time quantitative PCR for plasma cell induction experiment. **(B)** Bar plot illustrates the expression levels of B cell identity genes: BACH2, BCL6, and PAX5 in primary B cells and induced plasma cells harvested on day 11. A significant increase in BCL6 expression was observed in induced plasma cells, while a significant decrease was detected in the expression levels of BACH2 and PAX5. **(C)** A significant decrease in IRF4 expression was observed in induced plasma cells, whereas BLIMP1 expression showed a significant increase. **(D)** Bar chart illustrates the relative expression levels of total RUNX1 and RUNX1 isoforms (RUNX1a, RUNX1b, and RUNX1c) in primary B cells and induced plasma cells. A significant decrease in RUNX1c expression was observed in induced plasma cells, while no significant differences were detected in the expression levels of RUNX1a and RUNX1b. No significant differences were detected in the expression levels of XBP1. **(E-J)** Flow cytometrical analysis of B cell differentiation following siRNA transfection. CD19^+^ B cells were transduced twice with control-siRNA or RUNX-siRNA, before B cell activation and plasma cell induction. **(E)** Histogram shows RUNX1 expression in the primary B cells at different days after two rounds of treatment with control or RUNX1 siRNA silencing. Blue histogram: Control-siRNA, red histogram: RUNX1-siRNA. **(F)** Bar chart and trendline show the ratio of RUNX1 MFI reduction in RUNX1-siRNA treated cells over control cells. **(G)** Flow cytometrical data shows the percentages of CD138^+^ FSC^high^ cells among CD19^+^-gated cells at different days after induction. **(H)** Chart illustrates the percentages of CD138^+^ FSC^high^ cell at different days following induction in the presence of control or RUNX1 silencing. **(I)** Flow cytometrical data shows the percentages of CD138^+^ FSC^high^ plasma cells among CD19^+^ CD27^+^ CD38^+^-gated cells at different days after induction. **(J)** Chart illustrates the percentages of differentiating B among CD19^+^ CD27^+^ CD38^+^-gated cells in the presence of control or RUNX1 silencing. Statistical analysis was performed using a Student’s *t*-test (*P<0.05, **P < 0.01). For **(E, G)**, comparisons were made between RUNX1 knockdown and control samples at each time point of analysis.

To investigate the role of the RUNX1 gene in plasma cell differentiation during B cell development, we established an *in vitro* plasma cell induction system by stimulating primary B cells for 11 days in the presence of CD40L, IL-4 and IL-21 (**Figure 5A**). In our experimental setting, the sorted primary CD19^+^ cell population comprised over 70% naïve B cells and approximately 20% memory B cells. Flow cytometric analysis revealed an increase in plasma cell phenotype (CD138^+^ FSC^high^) across different stimulation time points. To further validate the identity of the induced plasma cells, qRT-PCR was performed. Among the B cell identity genes examined, *BACH2* and *PAX5* were significantly downregulated in the induced plasma cells, while *BCL6* was upregulated at 1.5-fold (**Figure 5B**). Additionally, plasma cell identity genes *BLIMP1* and *XBP1* were significantly upregulated in the induced plasma cells. However, *IRF4* showed downregulation in the induced plasma cells (**Figure 5C**). When RUNX1 level was examined, both *RUNX1a* and *RUNX1b* isoforms were upregulated, accompanied by decreased *RUNX1c* isoform (**Figure 5D**). The total RUNX1 level was elevated, but not statistically significant, in induced plasma cells compared to naïve B cells, consistent with observations in the human system.

RUNX1 was transiently knocked down using siRNA in B cells prior to plasma cell induction. Both control and RUNX1-targeting siRNA were administered twice over a 48-hour period to human CD19^+^ B cells, and cells were subsequently analyzed at multiple time points (**Figure 5A**). The efficiency of RUNX1 knockdown was assessed by flow cytometric analysis, which showed that RUNX1 suppression was evident on day 0 but subsided at day 3 (**Figures 5E, F**). No detectable suppression of RUNX1 was observed from day 8 onwards. Specifically, RUNX1 MFI decreased by a ratio of 0.13 ± 0.05 (corresponding to a reduction of 56 ± 27 MFI units) on day 0 compared to the control. By day 3, the ratio of RUNX1 reduction had declined to 0.08 ± 0.04 (reduction of 22 ± 12 MFI units), while reductions on subsequent days were minimal.

The percentages of differentiating B cells, as shown by CD19^+^ FSC^high^ CD138^+^ population, increased gradually from day 3 to day 11 in the control-siRNA treated cells (**Figures 5G, H**). Notably, cells transfected with RUNX1-targeting siRNA exhibited a slower rise in the percentages of CD19^+^ FSC^high^ CD138^+^ population. On day 3, the proportion of differentiating B cells was significantly lower in RUNX1-siRNA-treated cells (12.6 ± 0.4%) compared to control (17.2 ± 2.9%; P = 0.0130). However, no significant differences were observed at later time points: 19.9 ± 1.6% vs. 22.0 ± 2.3% on day 9, and 41.4 ± 2.5% vs. 41.0 ± 1.2% on day 11 for RUNX1- and control-siRNA treated cells, respectively. This short-term effect was likely due to the transient nature of siRNA-mediated knockdown, which typically lasts for approximately 3 days. Notably, the gated CD19^+^ FSC^high^ CD138^+^ population also displayed CD38^+^ and CD27^+^ phenotype, indicative of their activation capacity (**Supplementary Figure S5**).

To further confirm the lineage identity of the FSC^high^ CD138^+^ population as differentiating B cells, we analyzed their proportion within the CD19^+^ CD38^+^ CD27^+^-gated cells. The percentage of CD19^+^ CD38^+^ CD27^+^ FSC^high^ CD138^+^ cells increased progressively over the 11-day stimulation period (**Figures 5I, J**). RUNX1-siRNA–treated cells consistently showed a slower increase in differentiating B cell percentages, with a lower value at day 3 compared to the control (28.8 ± 4.8%, P = 0.0602). This trend persisted through days 8 and 11, although the differences did not reach statistical significance.

## Discussions

This study provides novel insights into the phenotypic landscape and molecular regulation of B cell differentiation in MM, with a particular focus on the dynamics of plasma cell populations and the expression of RUNX1 transcription factor. The immunophenotyping data reveal a significant expansion of CD19^int^ FSC^high^ CD20^-^ CD27^+^ CD38^+^ CD138^high^ plasma cells in the bone marrow of MM patients, consistent with the pathognomonic accumulation of malignant plasma cells in this hematological malignancy. Notably, while peripheral blood showed similar trends, these changes did not reach statistical significance, likely reflecting the bone marrow-centric nature of MM pathology. Human bone marrow plasma cells represent a heterogeneous population comprising distinct subsets, from newly arrived antibody-secreting cells to long-lived plasma cells, characterized by unique surface markers and transcriptomic profiles (34). The classification of plasma cell subpopulations further uncovered elevated proportions of pre-plasma and plasma cells in MM bone marrow. These findings are aligned with the notion that MM arises from aberrant plasma cell differentiation and suggest that both pre-terminal and terminal plasma cell states are dysregulated in MM.

Linear regression analysis showed no significant correlations between age and naïve B cells, memory B cells, activate B cells, pre-plasmablasts, plasmablasts, or plasma cells in MM patients. These findings suggest that the distribution of circulating B cell subsets remains relatively stable within the studied age range. Although aging can impact immune function through reduced B cell lymphopoiesis and repertoire diversity, the relative proportions of peripheral B cell subsets may not be affected (35–37). Therefore, the observed differences in B cell subset composition in MM patients are unlikely to be confounded by age-related changes.

RUNX1, also known as AML1, is a critical hematopoietic inducer in hematopoiesis. Loss of RUNX1 impaired the development of CD43^+^ and CD235^–^CD45&^+^ hematopoietic cells (38). RUNX1 and its family members also play roles in lymphocyte development and differentiation (21, 39). A key highlight of this study is the progressive upregulation of RUNX1 expression during B cell differentiation. RUNX1 levels were low in naïve, memory, and activated B cells, but increased substantially from the plasmablast stage and remained elevated in both pre-plasma and plasma cells. This stepwise increase pattern was consistent in both bone marrow and peripheral blood, suggesting that RUNX1 may play a crucial role in supporting or sustaining late-stage B cell differentiation. Notably, RUNX1 expression was significantly elevated in plasmablasts derived from MM patients compared to those from healthy donors. Interestingly, the consistently elevated expression of RUNX1 in plasmablasts at early stages raises the possibility of its involvement in early event in MM disease evolution. Furthermore, higher RUNX1 levels observed in RRMM patients, despite stable overall expression across ISS stages, suggests that RUNX1 may be reactivated or maintained during disease recurrence, possibly contributing to reshaping of transcriptional networks to favor tumor persistence. Besides, RUNX1 interaction with lenalidomide-sensitive pathways via interacting with Ikaros family zinc finger proteins (IKZF), further supports its involvement in relapse biology (40). This is consistent with studies showing that RUNX1 contributes to hematologic malignancies by enhancing abnormal proliferation and survival (19, 20). Interestingly, RUNX1 can be toggled between tumor suppressor and a classical oncogene, by synergistic interaction with the internal tandem duplications (ITDs) in the FLT3 receptor tyrosine kinase programs (41). These findings underscore the need to investigate RUNX1 expression longitudinally, including in premalignant samples, to determine whether its upregulation precedes full malignant transformation and contributes to relapse or therapeutic failure.

Although overall RUNX1 levels in bone marrow plasma cells did not differ significantly between MM patients and controls, the marked upregulation in the plasmablast subset suggests a potential pathogenic role for RUNX1 at an earlier stage of malignant differentiation. These findings support the notion that RUNX1 plays a key regulatory role in plasma cell differentiation. The progressive upregulation of RUNX1 across transitional B cell subsets suggests its involvement in reinforcing the plasma cell transcriptional program. This is consistent with previous studies demonstrating that lineage-defining transcription factors are dynamically regulated during B cell maturation to coordinate exit from the germinal center and acquisition of plasma cell identity (42, 43). RUNX1 may function in concert with other regulators such as BLIMP1 and XBP1 to stabilize the terminal differentiation state, highlighting its potential role not only as a marker but as an active driver of plasmacytic fate. This hypothesis was supported by estimation plot analysis, which confirmed higher RUNX1 expression in plasma cells compared to naïve B cells in each individual.

RUNX1 exists in three major isoforms, i.e. RUNX1a, RUNX1b, and RUNX1c, which are generated through alternative promoter usage and differential splicing. RUNX1a is the shortest isoform, composed primarily of the Runt DNA-binding domain but lacking the C-terminal transactivation domain, allowing it to act as a dominant-negative regulator of full-length RUNX1 isoforms (44, 45). In contrast, RUNX1b and RUNX1c are longer isoforms that possess both the DNA-binding and transactivation domains, enabling them to activate transcription of target genes involved in cell proliferation, differentiation, and survival. Among these isoforms, RUNX1b is most abundantly expressed in both T and B lymphocytes and is considered the predominant functional isoform during lymphoid development (32, 46, 47). RUNX1c, which is transcribed from a distal promoter, is also expressed in lymphocytes but is more restricted and typically induced during late stages of hematopoietic differentiation, particularly in thymocytes and activated B cell. RUNX1a, while detectable in early progenitor cells, is less expressed in mature lymphocytes and is believed to play a regulatory role by modulating the activity of the longer isoforms.

Given that the highest RUNX1 expression was observed in pre-plasma and plasma cell populations, and not in other B cell subsets or non-B cells, it is plausible that RUNX1 functions as a lineage-defining transcription factor rather than a marker of clonal evolution or disease severity. This is consistent with the observed upregulation of RUNX1 during *in vitro* plasma cell differentiation and the delayed plasma cell formation following transient RUNX1 knockdown, further supporting its role in promoting or stabilizing the plasma cell phenotype. However, while the lack of stage-specific differences reduces the likelihood of RUNX1 being a biomarker for disease progression or relapse, it does not preclude its involvement in MM pathogenesis. For instance, RUNX1 may act as a permissive factor required for the establishment of the malignant plasma cell state, but once this identity is acquired, its expression may plateau and remain stable across disease stages. Furthermore, the potential influence of the bone marrow microenvironment, genetic mutations, or post-translational modifications on RUNX1 function remains unexplored. Interestingly, RUNX1 and RUNX3 interact with Ikaros family zinc finger protein 1 (IKZF1) and IKZF3 to prevent their lenalidomide-induced degradation, and inhibiting RUNX proteins enhances immunomodulatory imide drugs (IMiD) sensitivity in multiple myeloma, offering a promising strategy to overcome drug resistance (40). Future investigations using larger, genomically stratified cohorts and functional models are needed to dissect whether RUNX1 collaborates with oncogenic pathways, such as MYC, to support MM cell survival or immune evasion. RUNX1 has an oncogenic role in T-cell acute lymphoblastic leukemia by altering Myb and Myc enhancer activity (48). Therefore, longitudinal studies assessing RUNX1 expression from diagnosis through treatment and relapse may provide insight into its potential role in minimal residual disease or therapeutic resistance.

In the B cell lineage, RUNX1 has been shown to cooperate with other transcription factors, such as E2A and EBF1, to regulate key checkpoints in early B cell development and influence the balance between self-renewal and differentiation (49). Notably, recent findings demonstrate that RUNX1 also integrates external signals from the microenvironment to modulate chromatin accessibility at critical enhancer regions, suggesting a role in epigenetic priming of differentiation programs (50). These insights align with our observations that RUNX1 is upregulated during late B cell differentiation and may function as a pivotal regulator of plasmablast transition. Interestingly, study has shown that IgA production is markedly impaired in B cells lacking both RUNX2 and RUNX3 as the induction of α germline transcription by retinoic acid and transforming growth factor-beta1 (TGF-β1) is completely abrogated (51).

The functional role of RUNX1 in plasma cell differentiation was substantiated through *in vitro* siRNA-mediated knockdown experiments. Mechanistically, RUNX1 silencing led to a delayed B cell differentiation trajectory, as indicated by slower emergence of CD138^high^ FSC^high^ cells. Concurrently, the expression of canonical plasma cell transcriptional regulators such as BLIMP1 and XBP1 was upregulated, while B cell-associated genes like BACH2 and PAX5 were downregulated, indicating a loss of naïve B cell identity accompanied by the acquisition of plasma cell characteristics (52). While an early reduction in CD138^+^ plasma cells was observed, both RUNX1 expression and plasma cell differentiation gradually recovered over time. This rebound is likely attributable to the temporary or transient nature of siRNA-mediated knockdown. This highlights the key limitation of transient knockdown approaches in long-term differentiation assays and emphasizes the need for more durable gene silencing tools, such as CRISPR-Cas9-mediated gene editing or stable expression of short hairpin RNA (shRNA), to fully delineate the functional role of RUNX1 in the future. Besides, the two rounds of siRNA transfection within the first 48 hours caused severe cell disruption and death, potentially triggering a bystander activation effect and leading to activation of the remaining cells. Hence, the culture medium was replenished frequently every 2 days to remove dead cells and debris.

The observed reduction in CD138^+^ plasma cells following RUNX1 knockdown suggests a potential delay in B cell differentiation during the late stages of *in vitro* B cell differentiation. This finding supports the role of RUNX1 as a positive regulator of plasmablast and plasma cell development. Nevertheless, the observed decrease in plasma cell percentages could also be attributed to alternative mechanisms, such as impaired B cell proliferation or enhanced apoptosis, both of which remain plausible explanations. Unfortunately, detailed analyses to dissect these possibilities were not feasible in the current experimental setup due to extensive cell death following the transfection process, which limited cell recovery. To elucidate the precise role of RUNX1 in plasma cell differentiation, future studies employing optimized transfection protocols or alternative gene silencing strategies with lower cytotoxicity are warranted. Incorporating cell viability, proliferation, and apoptosis assays in subsequent investigations will be critical in distinguishing between defects in differentiation and survival, thereby strengthening the mechanistic understanding of RUNX1 function in B cell biology.

In conclusion, this study underscores a significant expansion of plasma cell populations in MM bone marrow, accompanied by dynamic changes in RUNX1 expression during late B cell differentiation. The elevated expression of RUNX1 in MM plasmablasts, coupled with delayed plasma cell formation upon RUNX1 knockdown, suggests that RUNX1 plays a critical regulatory role in the transition from B cells to plasma cells. Although RUNX1 levels were not stage-specific, its early induction may represent a key molecular event in plasma cell differentiation and possibly myelomagenesis. The persistence of RUNX1 expression across MM stages and isoform shifts in differentiating B cells underscore the need to investigate RUNX1 not merely as a marker of differentiation but as an active orchestrator of lineage-specific transcriptional landscapes in both normal and malignant plasma cell contexts. These findings provide a compelling rationale for further exploration of RUNX1 as a potential biomarker or therapeutic target in MM.

## Data Availability

The original contributions presented in the study are included in the article/**Supplementary Material**. Further inquiries can be directed to the corresponding author/s.

## References

[1] Rajkumar SV. Multiple myeloma: 2020 update on diagnosis, risk-stratification and management. Am J Hematol. (2020) 95:548–67. doi: 10.1002/ajh.25791, PMID: 32212178

[2] Moreau P Kumar SK San Miguel J Davies F Zamagni E Bahlis N. Treatment of relapsed and refractory multiple myeloma: recommendations from the international myeloma working group. Lancet Oncol. (2021) 22:e105–e18. doi: 10.1016/S1470-2045(20)30756-7, PMID: 33662288

[3] Walker BA Wardell CP Melchor L Hulkki S Potter NE Johnson DC. Intraclonal heterogeneity and distinct molecular mechanisms characterize the development of T (4,14) and T (11,14) myeloma. Blood. (2012) 120:1077–86. doi: 10.1182/blood-2012-03-412981, PMID: 22573403

[4] Chng WJ Dispenzieri A Chim CS Fonseca R Goldschmidt H Lentzsch S. Imwg consensus on risk stratification in multiple myeloma. Leukemia. (2014) 28:269–77. doi: 10.1038/leu.2013.247, PMID: 23974982

[5] Manier S Sacco A Leleu X Ghobrial IM Roccaro AM. Bone marrow microenvironment in multiple myeloma progression. J BioMed Biotechnol. (2012) 2012:157496. doi: 10.1155/2012/157496, PMID: 23093834 PMC3471001

[6] Isola I Braso-Maristany F Moreno DF Mena MP Oliver-Calders A Pare L. Gene expression analysis of the bone marrow microenvironment reveals distinct immunotypes in smoldering multiple myeloma associated to progression to symptomatic disease. Front Immunol. (2021) 12:792609. doi: 10.3389/fimmu.2021.792609, PMID: 34880879 PMC8646031

[7] Avet-Loiseau H. Ultra high-risk myeloma. Hematol Am Soc Hematol Educ Program. (2010) 2010:489–93. doi: 10.1182/asheducation-2010.1.489, PMID: 21239841

[8] Zhuang J Da Y Li H Han B Wan X Zhu T. Cytogenetic and clinical risk factors for assessment of ultra high-risk multiple myeloma. Leuk Res. (2014) 38:188–93. doi: 10.1016/j.leukres.2013.11.010, PMID: 24342807

[9] Nutt SL Hodgkin PD Tarlinton DM Corcoran LM. The generation of antibody-secreting plasma cells. Nat Rev Immunol. (2015) 15:160–71. doi: 10.1038/nri3795, PMID: 25698678

[10] Shi W Liao Y Willis SN Taubenheim N Inouye M Tarlinton DM. Transcriptional profiling of mouse B cell terminal differentiation defines a signature for antibody-secreting plasma cells. Nat Immunol. (2015) 16:663–73. doi: 10.1038/ni.3154, PMID: 25894659

[11] Odendahl M Mei H Hoyer BF Jacobi AM Hansen A Muehlinghaus G. Generation of migratory antigen-specific plasma blasts and mobilization of resident plasma cells in a secondary immune response. Blood. (2005) 105:1614–21. doi: 10.1182/blood-2004-07-2507, PMID: 15507523

[12] Yu YH Lin KI. Factors that regulate the generation of antibody-secreting plasma cells. Adv Immunol. (2016) 131:61–99. doi: 10.1016/bs.ai.2016.03.001, PMID: 27235681

[13] Ochiai K Maienschein-Cline M Simonetti G Chen J Rosenthal R Brink R. Transcriptional regulation of germinal center B and plasma cell fates by dynamical control of irf4. Immunity. (2013) 38:918–29. doi: 10.1016/j.immuni.2013.04.009, PMID: 23684984 PMC3690549

[14] Tang TF Chan YT Cheong HC Cheok YY Anuar NA Looi CY. Regulatory network of blimp1, irf4, and xbp1 triad in plasmacytic differentiation and multiple myeloma pathogenesis. Cell Immunol. (2022) 380:104594. doi: 10.1016/j.cellimm.2022.104594, PMID: 36081178

[15] Turner CA Jr. Mack DH Davis MM. Blimp-1, a novel zinc finger-containing protein that can drive the maturation of B lymphocytes into immunoglobulin-secreting cells. Cell. (1994) 77:297–306. doi: 10.1016/0092-8674(94)90321-2, PMID: 8168136

[16] Shaffer AL Lin KI Kuo TC Yu X Hurt EM Rosenwald A. Blimp-1 orchestrates plasma cell differentiation by extinguishing the mature B cell gene expression program. Immunity. (2002) 17:51–62. doi: 10.1016/s1074-7613(02)00335-7, PMID: 12150891

[17] Muto A Ochiai K Kimura Y Itoh-Nakadai A Calame KL Ikebe D. Bach2 represses plasma cell gene regulatory network in B cells to promote antibody class switch. EMBO J. (2010) 29:4048–61. doi: 10.1038/emboj.2010.257, PMID: 20953163 PMC3020649

[18] Ito Y Bae SC Chuang LS. The runx family: developmental regulators in cancer. Nat Rev Cancer. (2015) 15:81–95. doi: 10.1038/nrc3877, PMID: 25592647

[19] Kurokawa M Hirai H. Role of aml1/runx1 in the pathogenesis of hematological Malignancies. Cancer Sci. (2003) 94:841–6. doi: 10.1111/j.1349-7006.2003.tb01364.x, PMID: 14556655 PMC11160144

[20] Ichikawa M Asai T Chiba S Kurokawa M Ogawa S. Runx1/aml-1 ranks as a master regulator of adult hematopoiesis. Cell Cycle. (2004) 3:722–4. doi: 10.4161/cc.3.6.951, PMID: 15213471

[21] Wong WF Kohu K Chiba T Sato T Satake M. Interplay of transcription factors in T-cell differentiation and function: the role of runx. Immunology. (2011) 132:157–64. doi: 10.1111/j.1365-2567.2010.03381.x, PMID: 21091910 PMC3050439

[22] Blyth K Cameron ER Neil JC. The runx genes: gain or loss of function in cancer. Nat Rev Cancer. (2005) 5:376–87. doi: 10.1038/nrc1607, PMID: 15864279

[23] Komori T. Regulation of bone development and extracellular matrix protein genes by runx2. Cell Tissue Res. (2010) 339:189–95. doi: 10.1007/s00441-009-0832-8, PMID: 19649655

[24] Reis BS Rogoz A Costa-Pinto FA Taniuchi I Mucida D. Mutual expression of the transcription factors runx3 and thpok regulates intestinal cd4(+) T cell immunity. Nat Immunol. (2013) 14:271–80. doi: 10.1038/ni.2518, PMID: 23334789 PMC3804366

[25] Kohu K Ohmori H Wong WF Onda D Wakoh T Kon S. The runx3 transcription factor augments th1 and down-modulates th2 phenotypes by interacting with and attenuating gata3. J Immunol. (2009) 183:7817–24. doi: 10.4049/jimmunol.0802527, PMID: 19933870

[26] Chuang LSH Matsuo J Douchi D Bte Mawan NA Ito Y. Runx3 in stem cell and cancer biology. Cells. (2023) 12(3):408. doi: 10.3390/cells12030408, PMID: 36766749 PMC9913995

[27] Wong WF Nakazato M Watanabe T Kohu K Ogata T Yoshida N. Over-expression of runx1 transcription factor impairs the development of thymocytes from the double-negative to double-positive stages. Immunology. (2010) 130:243–53. doi: 10.1111/j.1365-2567.2009.03230.x, PMID: 20102410 PMC2878468

[28] Niebuhr B Kriebitzsch N Fischer M Behrens K Gunther T Alawi M. Runx1 is essential at two stages of early murine B-cell development. Blood. (2013) 122:413–23. doi: 10.1182/blood-2013-01-480244, PMID: 23704093

[29] Seo W Ikawa T Kawamoto H Taniuchi I. Runx1-cbfbeta facilitates early B lymphocyte development by regulating expression of ebf1. J Exp Med. (2012) 209:1255–62. doi: 10.1084/jem.20112745, PMID: 22665574 PMC3405506

[30] Wong WF Kohu K Nagashima T Funayama R Matsumoto M Movahed E. The artificial loss of runx1 reduces the expression of quiescence-associated transcription factors in cd4(+) T lymphocytes. Mol Immunol. (2015) 68:223–33. doi: 10.1016/j.molimm.2015.08.012, PMID: 26350416

[31] Wong WF Kohu K Nakamura A Ebina M Kikuchi T Tazawa R. Runx1 deficiency in cd4+ T cells causes fatal autoimmune inflammatory lung disease due to spontaneous hyperactivation of cells. J Immunol. (2012) 188:5408–20. doi: 10.4049/jimmunol.1102991, PMID: 22551552

[32] Challen GA Goodell MA. Runx1 isoforms show differential expression patterns during hematopoietic development but have similar functional effects in adult hematopoietic stem cells. Exp Hematol. (2010) 38:403–16. doi: 10.1016/j.exphem.2010.02.011, PMID: 20206228 PMC2854264

[33] Rajkumar SV Dimopoulos MA Palumbo A Blade J Merlini G Mateos MV. International myeloma working group updated criteria for the diagnosis of multiple myeloma. Lancet Oncol. (2014) 15:e538–48. doi: 10.1016/S1470-2045(14)70442-5, PMID: 25439696

[34] Duan M Nguyen DC Joyner CJ Saney CL Tipton CM Andrews J. Understanding heterogeneity of human bone marrow plasma cell maturation and survival pathways by single-cell analyses. Cell Rep. (2023) 42:112682. doi: 10.1016/j.celrep.2023.112682, PMID: 37355988 PMC10391632

[35] Frasca D Blomberg BB. Aging impairs murine B cell differentiation and function in primary and secondary lymphoid tissues. Aging Dis. (2011) 2:361–73., PMID: 22396888 PMC3295082

[36] Scholz JL Diaz A Riley RL Cancro MP Frasca D. A comparative review of aging and B cell function in mice and humans. Curr Opin Immunol. (2013) 25:504–10. doi: 10.1016/j.coi.2013.07.006, PMID: 23932400 PMC3816359

[37] Mehr R Melamed D. Reversing B cell aging. Aging (Albany NY). (2011) 3:438–43. doi: 10.18632/aging.100313, PMID: 21483035 PMC3117459

[38] Shah Z Wang C Ullah H You H Philonenko ES Regan OV. Runx1 is a key inducer of human hematopoiesis controlling non-hematopoietic mesodermal development. Stem Cells. (2025) 43(5):sxaf019. doi: 10.1093/stmcls/sxaf019, PMID: 40220285

[39] Voon DC Hor YT Ito Y. The runx complex: reaching beyond haematopoiesis into immunity. Immunology. (2015) 146:523–36. doi: 10.1111/imm.12535, PMID: 26399680 PMC4693896

[40] Zhou N Gutierrez-Uzquiza A Zheng XY Chang R Vogl DT Garfall AL. Runx proteins desensitize multiple myeloma to lenalidomide via protecting ikzfs from degradation. Leukemia. (2019) 33:2006–21. doi: 10.1038/s41375-019-0403-2, PMID: 30760870 PMC6687534

[41] Behrens K Triviai I Schwieger M Tekin N Alawi M Spohn M. Runx1 downregulates stem cell and megakaryocytic transcription programs that support niche interactions. Blood. (2016) 127:3369–81. doi: 10.1182/blood-2015-09-668129, PMID: 27076172

[42] Cobaleda C Schebesta A Delogu A Busslinger M. Pax5: the guardian of B cell identity and function. Nat Immunol. (2007) 8:463–70. doi: 10.1038/ni1454, PMID: 17440452

[43] Sciammas R Shaffer AL Schatz JH Zhao H Staudt LM Singh H. Graded expression of interferon regulatory factor-4 coordinates isotype switching with plasma cell differentiation. Immunity. (2006) 25:225–36. doi: 10.1016/j.immuni.2006.07.009, PMID: 16919487

[44] Real PJ Navarro-Montero O Ramos-Mejia V Ayllon V Bueno C Menendez P. The role of runx1 isoforms in hematopoietic commitment of human pluripotent stem cells. Blood. (2013) 121:5250–2. doi: 10.1182/blood-2013-03-487587, PMID: 23813937

[45] Guan L Voora D Myers R Del Carpio-Cano F Rao AK. Runx1 isoforms regulate runx1 and target genes differentially in platelets-megakaryocytes: association with clinical cardiovascular events. J Thromb Haemost. (2024) 22:3581–98. doi: 10.1016/j.jtha.2024.07.032, PMID: 39181539 PMC11608153

[46] Wong WF Looi CY Kon S Movahed E Funaki T Chang LY. T-cell receptor signaling induces proximal runx1 transactivation via a calcineurin-nfat pathway. Eur J Immunol. (2014) 44:894–904. doi: 10.1002/eji.201343496, PMID: 24310293

[47] Wong WF Kurokawa M Satake M Kohu K. Down-regulation of runx1 expression by tcr signal involves an autoregulatory mechanism and contributes to il-2 production. J Biol Chem. (2011) 286:11110–8. doi: 10.1074/jbc.M110.166694, PMID: 21292764 PMC3064165

[48] Choi A Illendula A Pulikkan JA Roderick JE Tesell J Yu J. Runx1 is required for oncogenic myb and myc enhancer activity in T-cell acute lymphoblastic leukemia. Blood. (2017) 130:1722–33. doi: 10.1182/blood-2017-03-775536, PMID: 28790107 PMC5639483

[49] Sood R Kamikubo Y Liu P. Role of runx1 in hematological Malignancies. Blood. (2017) 129:2070–82. doi: 10.1182/blood-2016-10-687830, PMID: 28179279 PMC5391618

[50] Thomsen I Kunowska N de Souza R Moody AM Crawford G Wang YF. Runx1 regulates a transcription program that affects the dynamics of cell cycle entry of naive resting B cells. J Immunol. (2021) 207:2976–91. doi: 10.4049/jimmunol.2001367, PMID: 34810221 PMC8675107

[51] Watanabe K Sugai M Nambu Y Osato M Hayashi T Kawaguchi M. Requirement for runx proteins in iga class switching acting downstream of tgf-beta 1 and retinoic acid signaling. J Immunol. (2010) 184:2785–92. doi: 10.4049/jimmunol.0901823, PMID: 20142360

[52] Shaffer AL Shapiro-Shelef M Iwakoshi NN Lee AH Qian SB Zhao H. Xbp1, downstream of blimp-1, expands the secretory apparatus and other organelles, and increases protein synthesis in plasma cell differentiation. Immunity. (2004) 21:81–93. doi: 10.1016/j.immuni.2004.06.010, PMID: 15345222

