# Supplementary material for: RUNX1 expression dynamics in plasma cell differentiation and pathogenesis of multiple myeloma
Source: Front Immunol. 2025 Sep 8;16:1643615. doi: 10.3389/fimmu.2025.1643615 (PMC12450927; doi:10.3389/fimmu.2025.1643615)
Supplement: Supplementary file 1 [file DataSheet1.pdf]

RUNX1 Expression Dynamics in Plasma Cell Differentiation and Pathogenesis of Multiple Myeloma

Ting Fang Tang [1], Yee Teng Chan [1], Hui Jing Lim [1], Nur Adila Anuar [2], Chin Sum Cheong [2], Chung Yeng Looi [3,4], Sen Mui Tan [5], Won Fen Wong [1,*], Gin Gin Gan [2,*]

Supplementary Data

Supplementary Figure S1. Linear regression analysis of age versus percentages of plasma cell subsets in control and MM bone marrow.

Supplementary Figure S2. Linear regression analysis of age versus percentages of plasma cell subsets in control and MM peripheral blood.

Supplementary Figure S3. Linear regression analysis of age versus RUNX expression in control or MM bone marrow plasma cell subsets.

Supplementary Figure S4. Linear regression analysis of age versus RUNX expression in control or MM bone marrow plasma cell subsets.

Supplementary Figure S5. Expression of CD38 and CD27 markers in the FSC^{high} CD138^{+}-gated cell population in in vitro plasma cell induction.

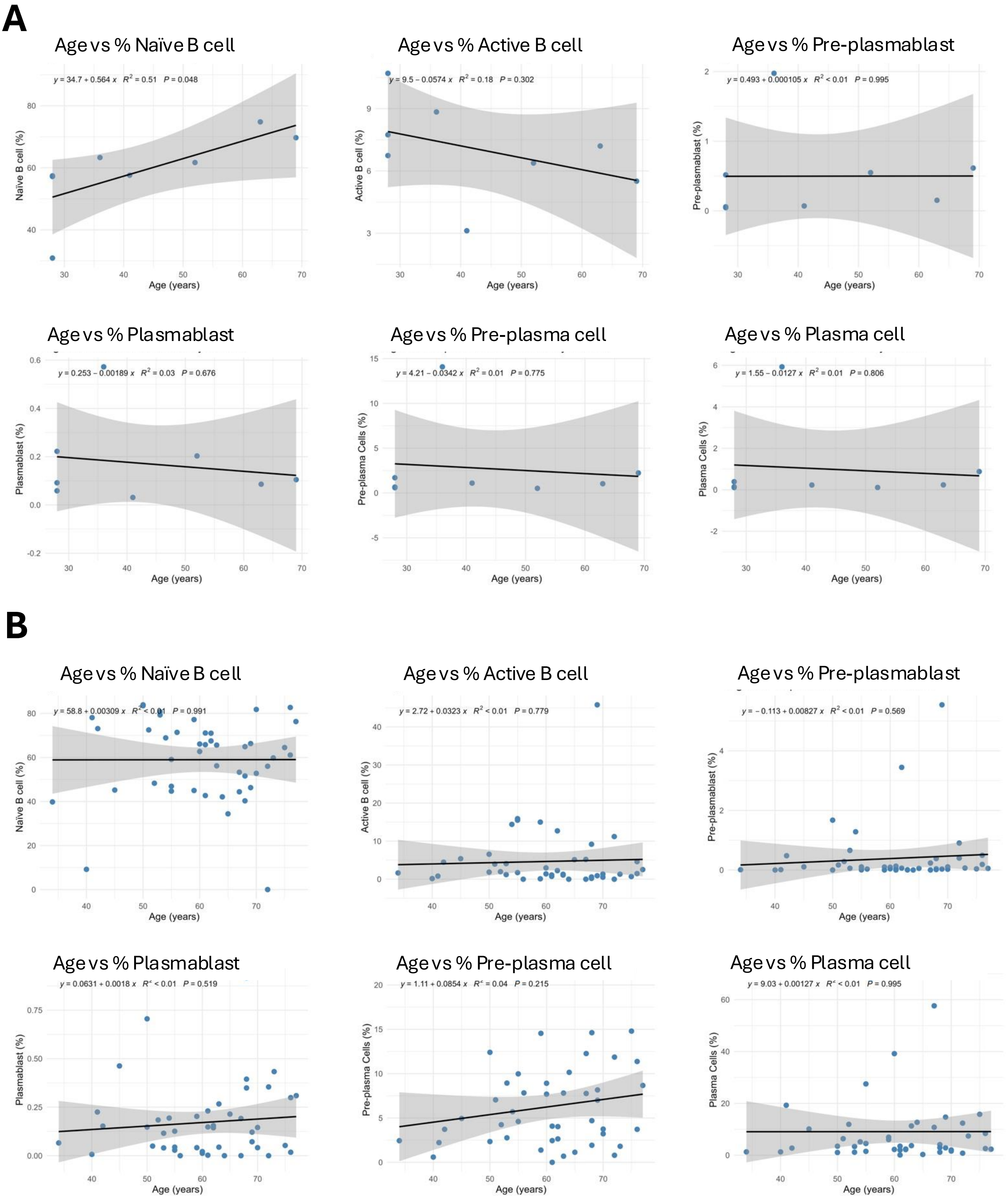

Supplementary Figure S1. Linear regression analysis of age versus percentages of plasma cell subsets in control and MM bone marrow.

Linear regression plots illustrating the correlation analysis between age of healthy controls (A) or MM patients (B) and the percentages of various B cell subsets, including naïve B, active B, pre-plasmablasts, plasmablasts, pre-plasma and plasma cells, in bone marrow. Each point represents an individual donor. The fitted regression line with 95% confidence bands is shown. No significant correlations were observed between age and the proportions of most B cell subsets, suggesting age-independent distribution in the studied cohort.

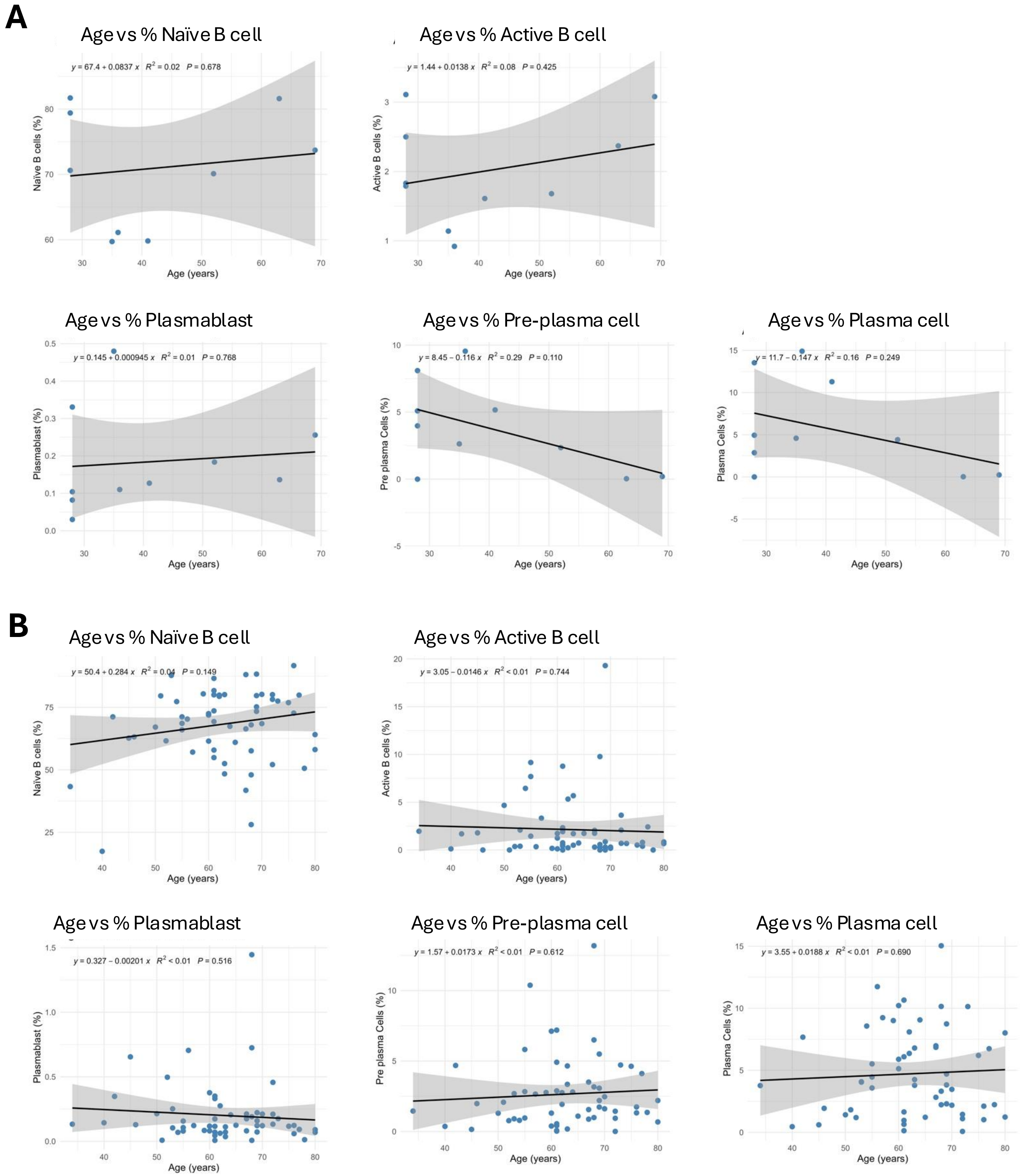

Supplementary Figure S2. Linear regression analysis of age versus percentages of plasma cell subsets in control and MM peripheral blood.

Linear regression plots illustrating the correlation analysis between age of healthy controls (A) or MM patients (B) and the percentages of various B cell subsets, including naïve B, active B, plasmablasts, pre-plasma and plasma cells, in peripheral blood. Each point represents an individual donor. The fitted regression line with 95% confidence bands is shown. No significant correlations were observed between age and the proportions of most B cell subsets, suggesting age-independent distribution in the studied cohort.

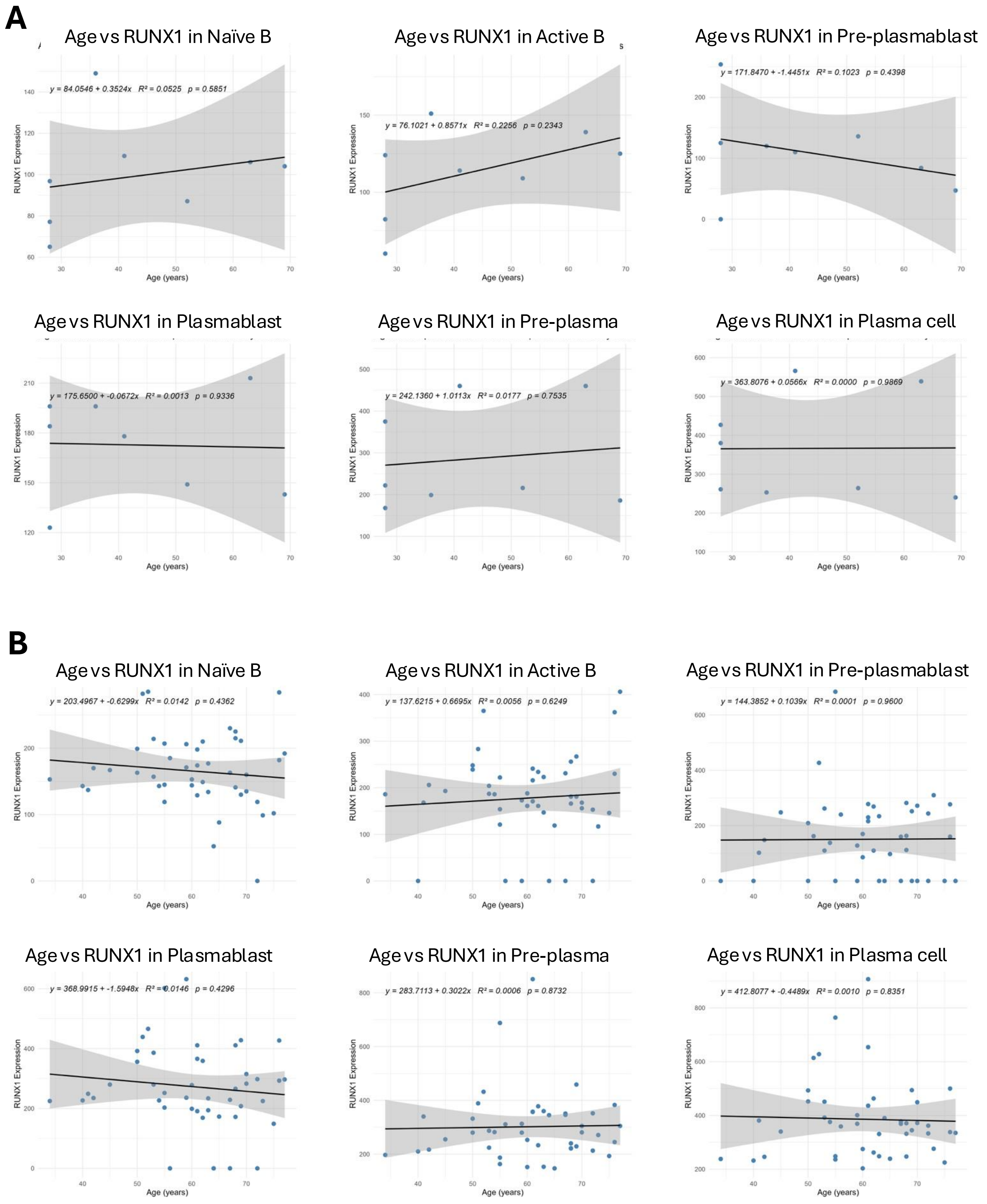

Supplementary Figure S3. Linear regression analysis of age versus RUNX expression in control or MM bone marrow plasma cell subsets.

Linear regression plots illustrating the correlation analysis between age of healthy controls (A) or MM patients (B) and the percentages of RUNX1 expression (MFI) in various B cell subsets, including naïve B, active B, pre-plasmablasts, plasmablasts, pre-plasma and plasma cells, in bone marrow. Each point represents an individual donor. The fitted regression line with 95% confidence bands is shown. No significant correlations were observed between age and RUNX1 expression level, suggesting age-independent distribution in the studied cohort.

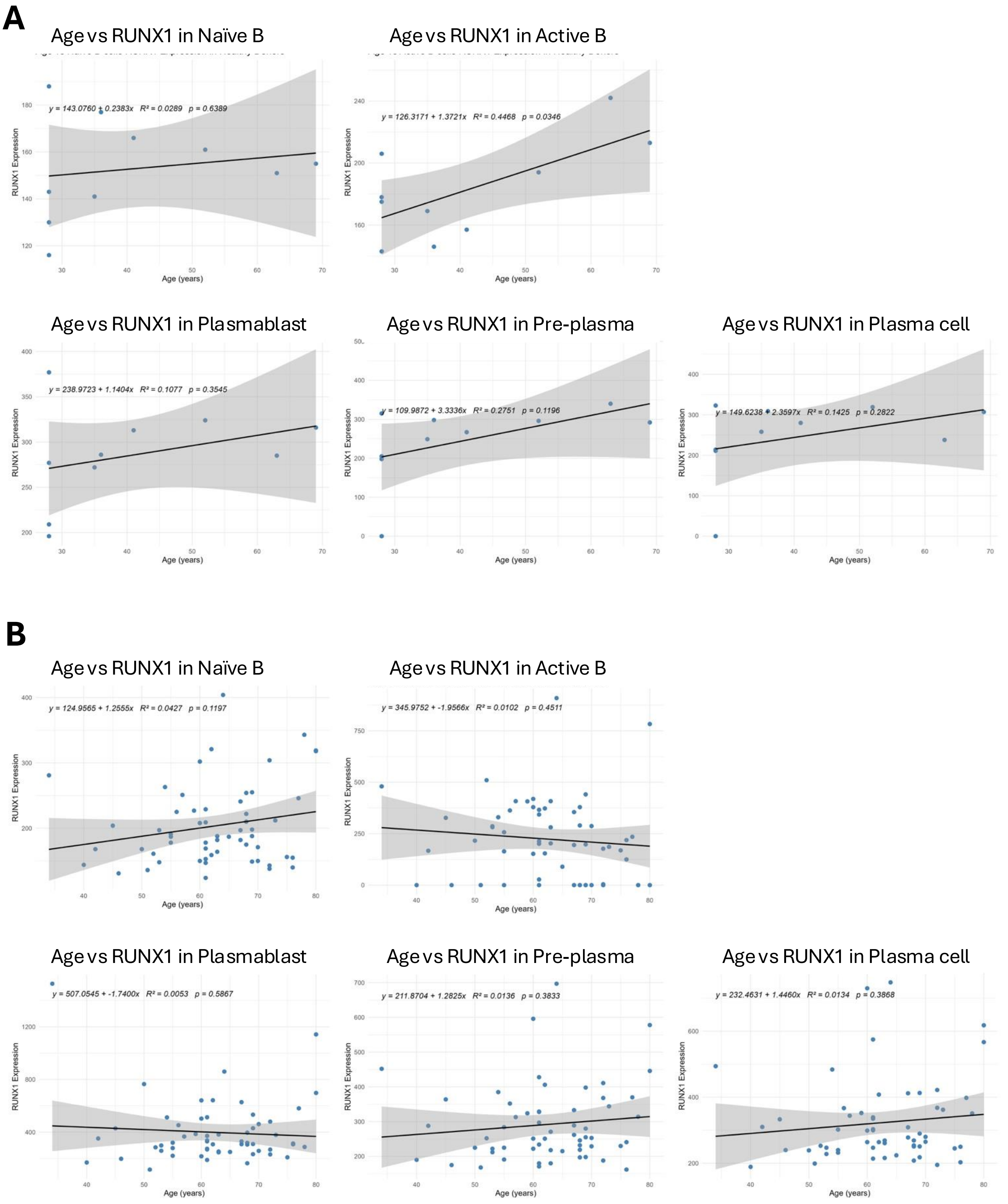

Supplementary Figure S4. Linear regression analysis of age versus RUNX expression in control or MM bone marrow plasma cell subsets.

Linear regression plots illustrating the correlation analysis between age of healthy controls (A) or MM patients (B) and the percentages of RUNX1 expression (MFI) in various B cell subsets, including naïve B, active B, plasmablasts, pre-plasma and plasma cells, in peripheral blood. Each point represents an individual donor. The fitted regression line with 95% confidence bands is shown. No significant correlations were observed between age and RUNX1 expression level, suggesting age-independent distribution in the studied cohort.

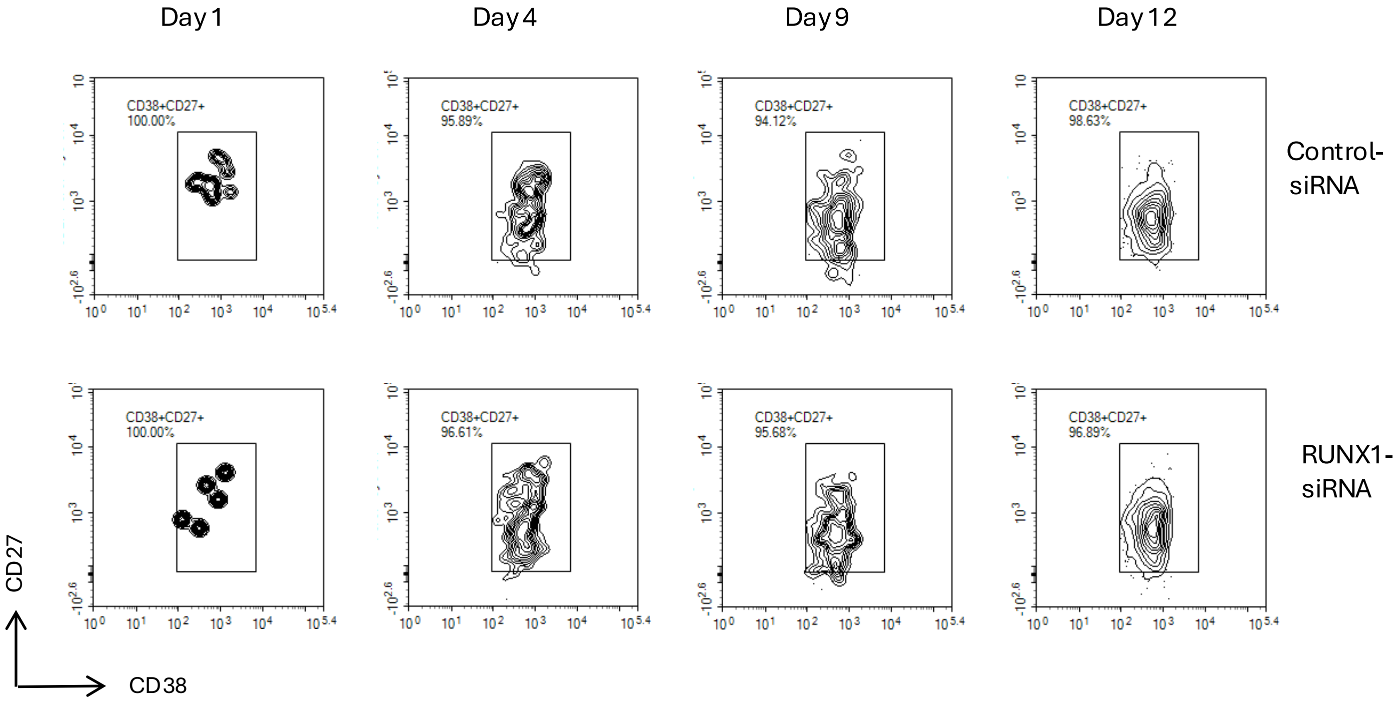

Supplementary Figure S5. Expression of CD38 and CD27 markers in the FSC^{high} CD138^{+}-gated cell population in in vitro plasma cell induction.

Primary CD19^{+} B cells were isolated and cultured in the presence of CD40L, IL-4 and IL-21 for 12 days to induce plasma cell differentiation in vitro, as described in the main text. Flow cytometrical data shows expression of CD38 and CD27 in the FSC^{high} CD138^{+}-gated cell population at different days of plasma cell differentiation process (Figure 5F). FSC^{high} CD138^{+}-gated cell showed CD38+, indicating robust cell activation and differentiation capacity.
